# Lowering the target daily light integrals following days with excessive lighting can reduce lettuce production costs

**DOI:** 10.3389/fpls.2024.1467443

**Published:** 2024-12-10

**Authors:** Andres M. Mayorga-Gomez, Marc W. van Iersel, Rhuanito Soranz Ferrarezi

**Affiliations:** Department of Horticulture, University of Georgia, 1111 Miller Plant Sciences, Athens, GA, United States

**Keywords:** *Lactuca sativa*, light levels, energy, supplemental light, daily light integral

## Abstract

Given the fluctuating availability of natural lighting throughout the year, supplemental light is frequently employed to maintain the optimal daily light integral (DLI) levels necessary for adequate plant growth. However, the use of supplemental light translates into higher operational costs. Recent reports suggest that plants can tolerate a day with low DLI following exposure to a day with high DLI from natural light. This was referred to as the ‘carryover’ effect. In such cases, supplemental lighting may not be necessary, resulting in energy savings. In this study, we determined if plants can withstand such DLI fluctuations over multiple days without compromising plant growth. Additionally, we calculated the energy requirements for trese treatments to evaluate the potential energy savings of the carryover effect. To test this, we cultivated lettuce plants (*Lactuca sativa* cv. ‘Waldmand’s Dark Green’ and ‘Rouxai’) in a walk-in grow chamber, subjecting them to six different lighting treatments. Each treatment consisted of a day with a high DLI of 22.5 mol·m^-2^·d^-1^ followed by a varying number of consecutive days with low DLI, ranging from 1 to 5 days, with DLIs of 7.5, 11.25, 12.5, 13.13, and 13.5 mol·m^-2^·d^-1^ respectively. The combined DLI for each treatment, calculated as the average DLI across high and low DLI days, was maintained at 15 mol·m^-2^·d^-1^. Additionally, we included a control treatment where plants were exposed to a constant DLI of 15 mol·m^-2^·d^-1^. We measured plant growth rate, final fresh and dry weights, leaf number, leaf area, specific leaf area, light use efficiency, and relative pigment content to assess differences in plant growth under the different lighting regimes. We observed a decrease in biomass accumulation, as indicated by a 13% reduction in final dry weight only for the treatment involving one day of high DLI followed by one day of low DLI, compared to our control. We discovered that plants can tolerate multiple days of low DLI following a day with high DLI, in contrast to the optimal values reported in the literature. This finding can lead to reduced energy consumption for supplemental lighting and consequent operational cost savings.

## 1 Introduction

Daily light integral (DLI) refers to the amount of photosynthetically active radiation provided to plants in 24 hours (Faust et al., 2005). DLI has been used as a reference point for the required light for optimal plant growth. Runkle (2019) reported a guideline specifying the essential DLI for cultivating different crops in controlled environments. However, the availability of light varies based on geographical locations since light levels differ significantly in the northern parts of the U.S. compared to southern locations. Additionally, there is less sunlight available in winter compared to summer overall. Supplemental lighting becomes essential to ensure consistent production throughout the year (Korczynski et al., 2022). Not only supplemental light is implemented to achieve DLIs reported to be optimal. For instance, Albright et al. (2000) developed a lighting control that includes shadings and supplemental light to maintain optimal DLI for plant growth. However, the cost for supplemental lighting in a vegetable greenhouse may amount to USD $200,000 per hectare, constituting 30% of the annual farm gate value (van Iersel and Gianino., 2017). Studies have focused on reducing electricity consumption and energy pricing associated with supplemental light. In 2017, van Iersel and Gianino (2017) proposed an adaptive control system for light-emitting diode (LED) lights. This system adjusts photosynthetic photon flux density (PPFD) levels by considering the natural sunlight to achieve the target DLIs. They claim electricity consumption reductions of 20-92%. In 2021, Bhuiyan and van Iersel (2021) conducted a study testing lettuce growth under fluctuating PPFD levels every 15 minutes. They observed that when these PPFD fluctuations were not extreme, lettuce plants exhibited normal growth without significant adverse effects. This suggests that lettuce can tolerate fluctuations in PPFD levels, potentially leading to energy cost savings if paired with variable electricity prices.

Most recently, Jayalath et al. (2024). reported that plants can grow unaffectedly with extended lighting fluctuations. They conducted experiments growing lettuce in greenhouses and indoor conditions (growth chamber), varying the target DLI from one day to the next. The DLI fluctuation between two consecutive days ranged from 5 to 25 mol·m^-2^·d^-1^. Interestingly, plants exposed to DLI fluctuations with a difference of 10 mol·m^-2^·d^-1^ showed no negative effects on their growth. They propose the existence of a ‘carryover’ effect from days with high DLI (exceeding the target) to days with low DLI (falling below the target). This suggests that in agriculture setups utilizing supplemental light, it might not be always necessary to reach a specific DLI for lettuce if plants get exposed to a higher DLI than the target the day before, generating a reduction in electricity consumption.

The present study assessed whether plants can tolerate DLI fluctuations over consecutive days without compromising growth in an indoor growth chamber. Specifically, our goal was to investigate the viability of the ‘carryover’ effect for plants exposed to a high DLI for one day, followed by multiple days of a low DLI (falling below the target DLI). If plants can tolerate such lighting fluctuations, potential significant electricity savings exist. Additionally, we determined the energy requirements for trese treatments to evaluate the potential energy savings of the carryover effect.

## 2 Results

### 2.1 Plant growth rate

**Figure 1:**
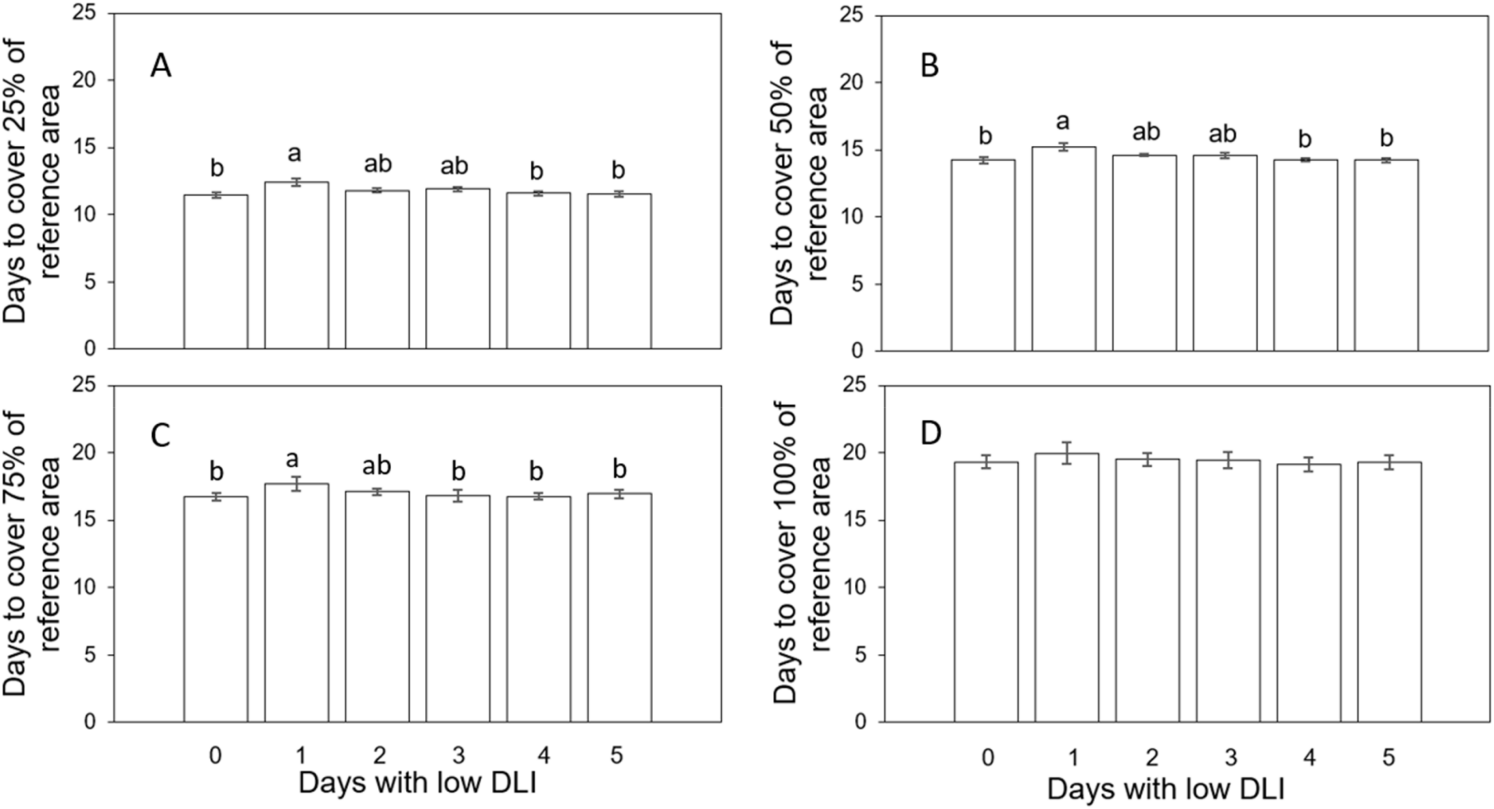
Number of days that plants growing under different daily light integral (DLI) treatments needed to achieve the coverage of a reference area of 25% **(A)**, 50% **(B)**, 75% **(C)**, and 100% **(D)**. Error bars denote standard error (n=6). Different letters on top of error bars show significant differences at α=0.05 from Tukey’s test for **A, B**. Different letters on top of error bars show significant differences at α=0.05 from Fisher’s LSD test for **(C)** Absence of letters on top of error bars show no significant differences.

We determined the time needed to reach different coverage levels in a specific area to analyze plant growth rates by estimating the days based on the sigmoidal curves fitted to the projected canopy size (PCS) information. Plants growing under different lighting regimes needed different days to achieve specific sizes indicated by the coverage percentage of a reference area. For example, to cover 25% of the said area, plants growing with 0, 1, 2, 3, 4, and 5 days with low DLI following a day with high DLI needed an average of 11.46, 12.41, 11.81, 11.86, 11.6, and 11.54 days, respectively (**Figure 1A**). We did not find a significant interaction between the lettuce cultivars and lighting treatment (F_5,24_ = 0.028, *P*=0.99). The time taken to achieve this level of coverage showed significant differences (F_5,29_ = 3.91, *P*=0.007) between plants growing with one day of low DLI compared to the control treatment (*P*=0.007) (0 days with low DLI) and plants with 4 (*P*=0.029) and 5 (*P*=0.017) days with low DLI.

Likewise, to cover 50% of the reference area, plants subjected to 0, 1, 2, 3, 4 and 5 days with low DLI following a day with high DLI needed 14.25, 15.22, 14.58, 14.6, 14.26, and 14.24 days, respectively (**Figure 1B**). The interaction between cultivar and lighting treatment was not significant (F_5,24_ = 0.26, P=0.92). Once again, the time needed to attain this coverage level exhibited significant differences (F_5, 29_ = 3.62, P=0.011) when comparing plants subjected to one day of low DLI when compared with the control (*P*=0.017) and plants with 4 (*P*=0.023) and 5 (*P*=0.018) days with low DLI.

To achieve 75% coverage, plants growing with 0, 1, 2, 3, 4 and 5 days with low DLI following a day with high DLI needed 16.75, 17.68, 17.1, 16.97, 16.76, and 16.96 days, respectively (**Figure 1C**). Here, significant differences were found (F_5,29_ = 2.59, *P*=0.046) (Tukey’s test did not show differences when comparing means) with no interaction between lighting treatments and cultivars (F_5,24_ = 0.9, *P*=0.49). Since Tukey’s test did not show differences when comparing means, we used Fisher’s LSD test. Here, plants growing with one day of low DLI needed significantly more days to achieve 75% of coverage when compared to plants growing under 0 (*P*=0.005), 3 (*P*=0.0087), 4 (*P*=0.0064) and 5 (*P*=0.028) days with low DLI.

Finally, to cover 100% of the reference area, plants growing under 0, 1, 2, 3, 4 and 5 days with low DLI following a day with high DLI needed 19.3, 19.96, 19.5, 19.72, 19.14, and 19.27 days, respectively (**Figure 1D**). There were no significant differences among treatments regarding covering the equivalent of the entire reference area (F_5,29_ = 0.76, *P*=0.26) (F_5,29_ = 1.35, *P*=0.26). Interaction between cultivar and lighting treatments was not found (F_5,24_ = 1.35, *P*=0.29). (F_5,24_ = 1.304, *P*=0.29).

### 2.2 Leaf area and specific leaf area

**Figure 2:**
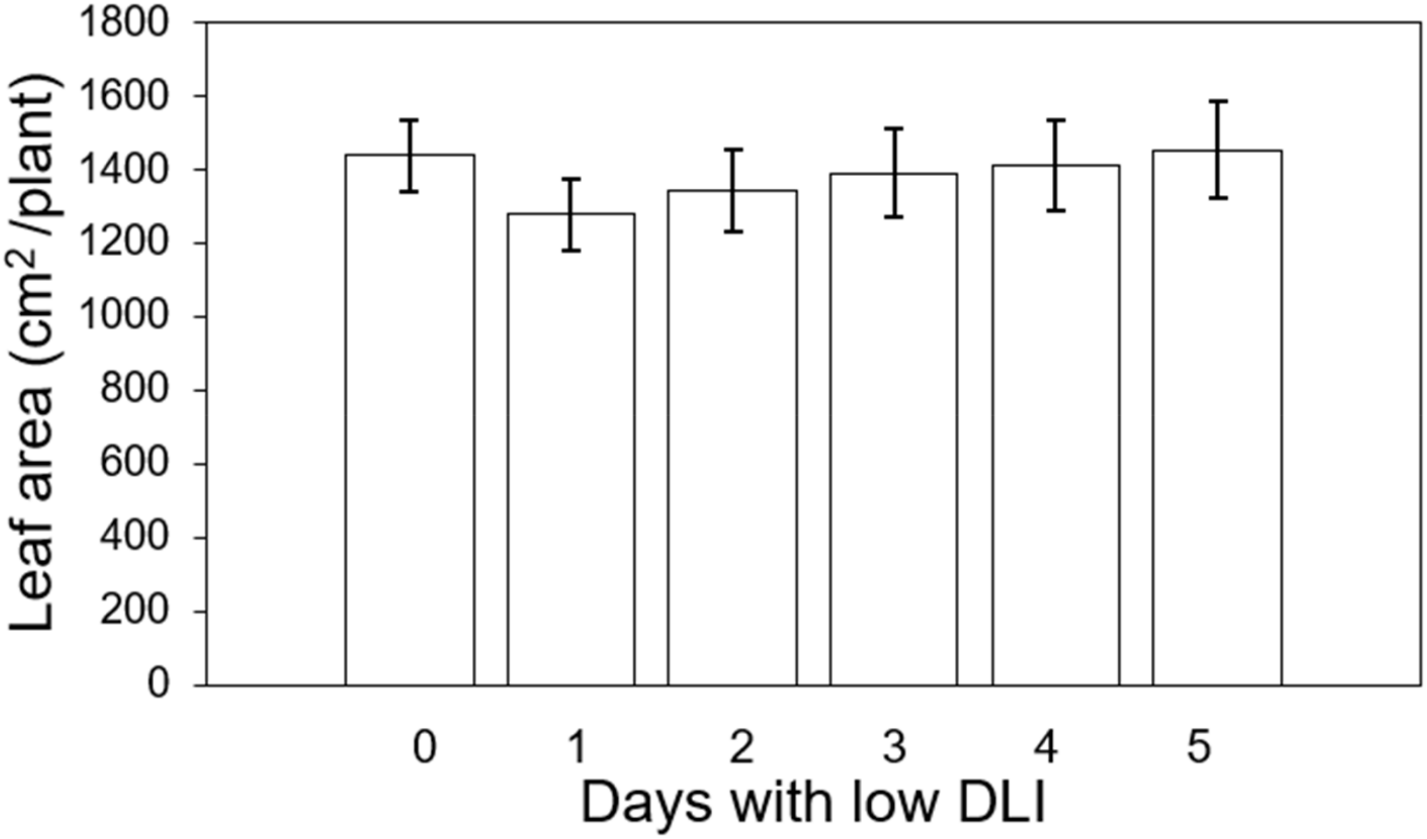
Final leaf area of ‘Rouxai’ and ‘Walmand’s dark green’ lettuce plants growing under different lighting treatments. Lighting treatments are described by the number of days with low daily light integral (DLI) after a day with high DLI. Error bars denote standard error (n=6). Absence of letters on top of error bars show no significant differences at α=0.05 from Tukey’s test.

We measured the final leaf area on three plants situated at the central transverse position of each tray, yielding a total count of six plants. The average leaf area for plants growing under the least amount of days with low DLI (T0) ascendingly to 5 days with DLI was as follows: 1436.09, 1276.86, 1342.12, 1388.37, 1409.01 and 1452.79 cm^2^ respectively (**Figure 2**). However, we did not find significant differences in this parameter (F_5,29_ = 1.65, *P*=0.17). Furthermore, there was no interaction between light treatment and cultivar (F_5,24_ = 0.36, *P*=0.88).

**Figure 3:**
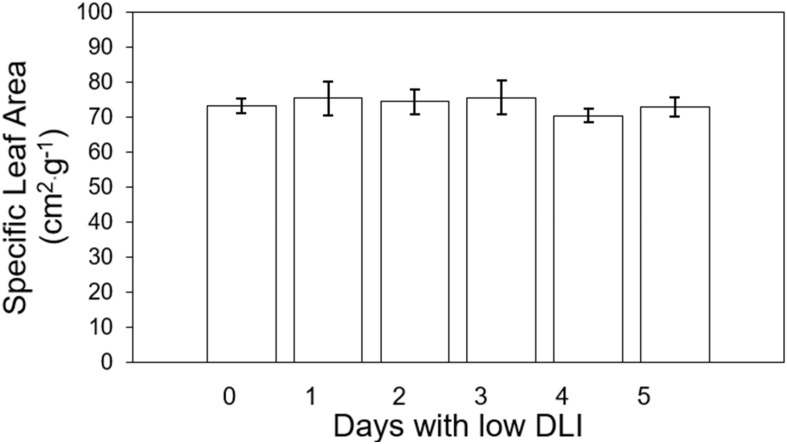
Specific leaf area of ‘Rouxai’ and ‘Walmand’s dark green’ lettuce plants growing under different lighting treatments. Specific leaf area was calculated as the ratio of final leaf area and final fry weight. Lighting treatments are described by the number of days with low daily light integral (DLI) after a day with high DLI. Error bars denote standard error (n=6). Absence of letters on top of error bars show no significant differences at α=0.05 from Tukey’s test.

We did not find significant differences (F_5,29_ = 0.79, *P*=0.56) in specific leaf area (SLA) among plants growing under different lighting treatments, and there was no interaction between SLA and lettuce cultivar (F_5,24_ = 0.74, *P*=0.59). The average SLA per plant from treatment 0 to 5 days were as follows: 407.86, 371.18, 386.29, 370.17, 355.63, and 370.17 cm^2^·g^-1^ (**Figure 3**).

### 2.3 Pigment content

**Figure 4:**
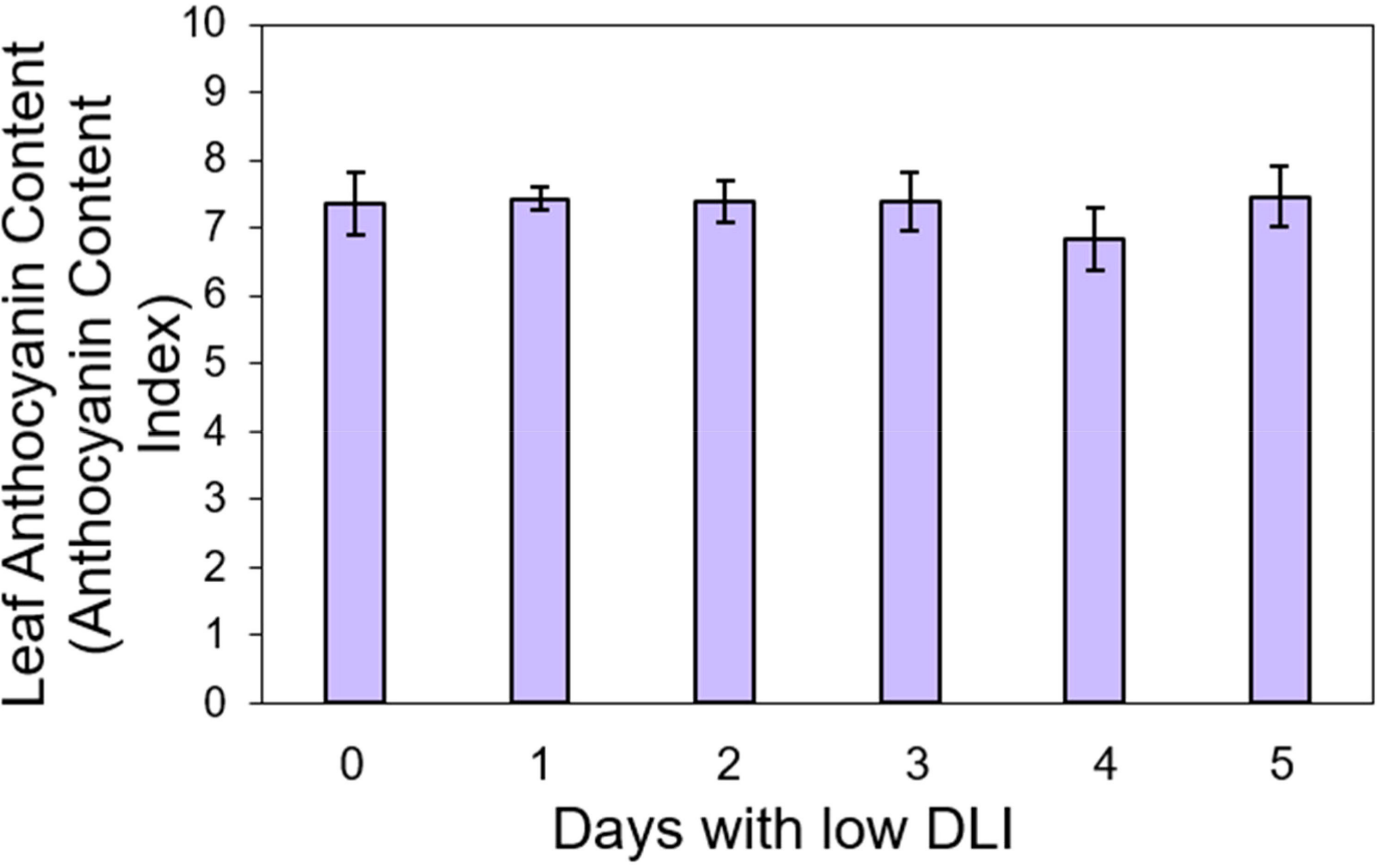
Leaf anthocyanin content of ‘Rouxai’ lettuce plants growing under different lighting treatments. Lighting treatments are described by the number of days with low daily light integral (DLI) after a day with high DLI. Error bars show standard error (n=3). Absence of letters on top of error bars show no significant differences at α=0.05 from Tukey’s test.

**Figure 5:**
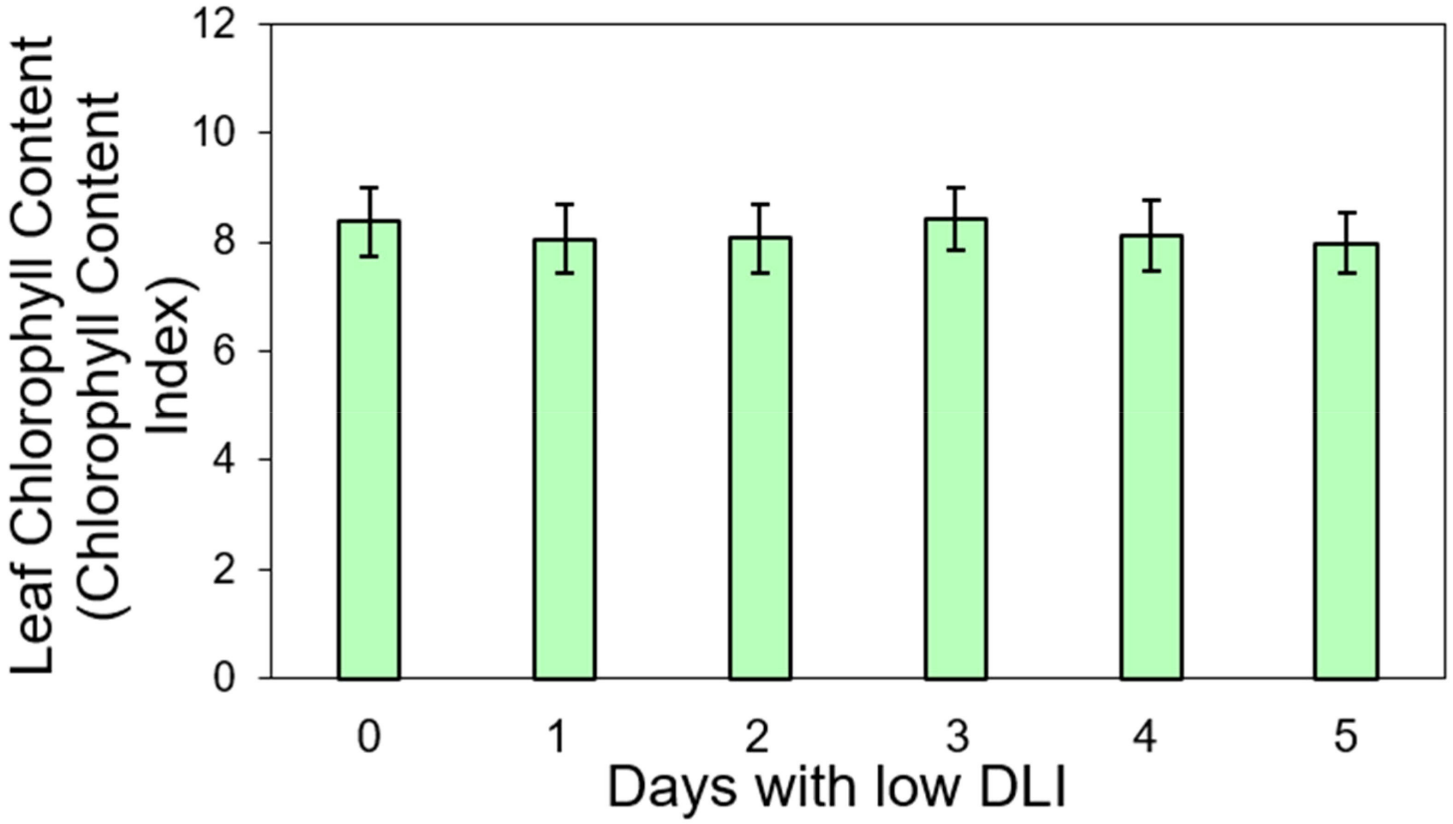
Leaf chlorophyll content in ‘Walmand’s dark green’ and ‘Rouxai’ plants (both cultivars averaged) growing under different lighting treatments. Lighting treatments are described by the number of days with low daily light integral (DLI) after a day with high DLI. Error bars show standard error (n=6). Absence of letters on top of error bars show no significant differences at α=0.05 from Tukey’s test.

We conducted pigment content measurements on both lettuce varieties. For ‘Rouxai’, the leaf anthocyanin content varied from 7.36 to 7.46 anthocyanin content index (ACI) across treatments with varying low DLI days (**Figure 4**), with no significant differences found (F_5,12_ = 0.36, *P*=0.86). Additionally, the leaf chlorophyll content in sequence from treatment T0 to T5 were 8.36, 8.05, 8.06, 8.41, 8.11, and 7.98 chlorophyll content index (CCI) (**Figure 5**). However, no significant differences were found (F_5,29_ = 1.02, *P*=0.42) nor interaction between cultivar and treatment (F_5,29_ = 0.74, *P*=0.59).

### 2.4 Shoot dry weight

**Figure 6:**
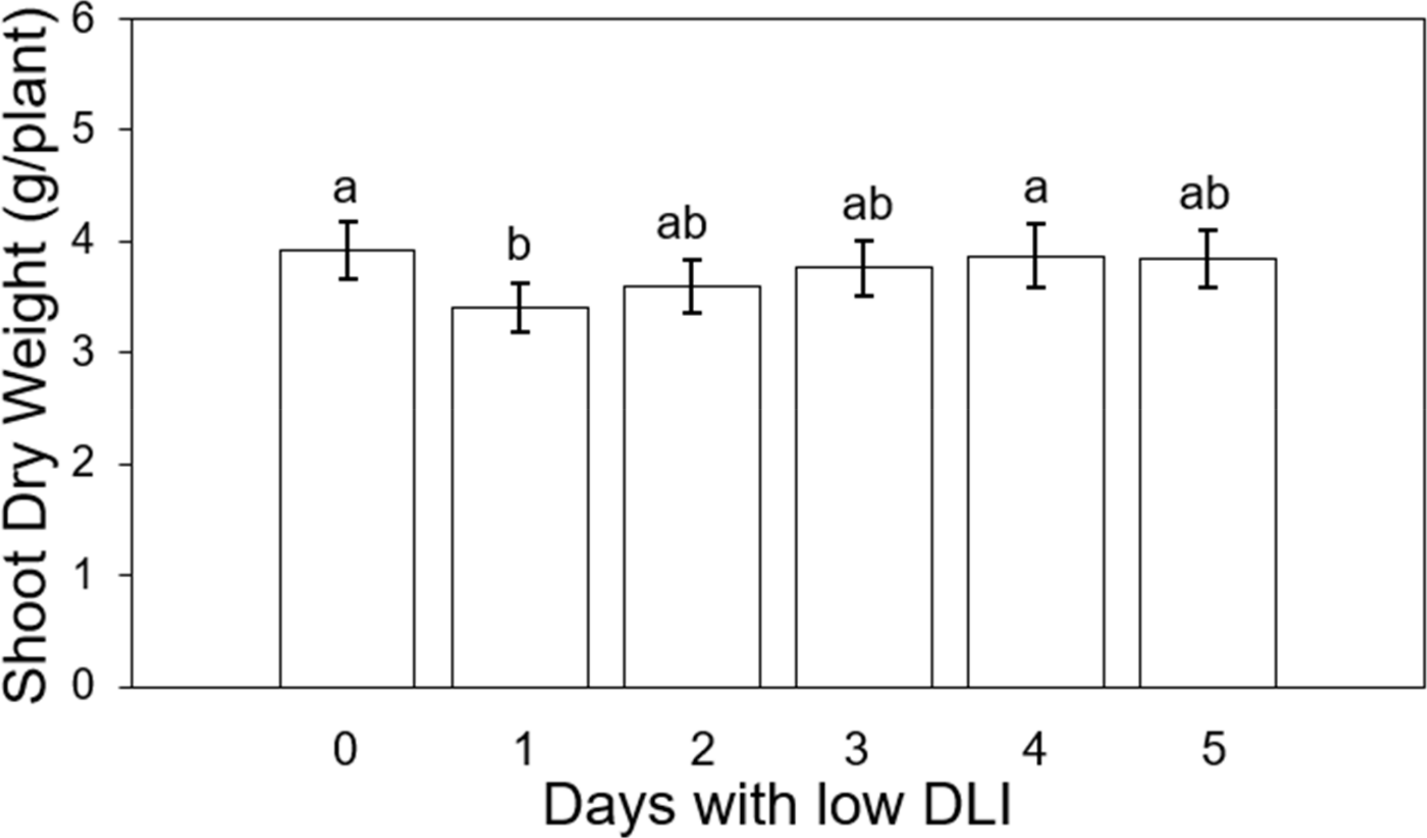
Final shoot dry weight of ‘Rouxai’ and ‘Walmand’s dark green’ lettuce plants growing under different lighting treatments. Lighting treatments are described by the number of days with low daily light integral (DLI) after a day with high DLI. Error bars denote standard error (n=6). Different letters on top of error bars show significant differences at α=0.05.

We averaged the final shoot dry weight for the different lighting treatments for both cultivars. The values in sequence from treatment T0 to T5 were recorded as follows: 3.91, 3.4, 3.6, 3.75, 3.87, and 3.83 g per plant (**Figure 6**). The number of days of low DLI significantly affected the final shoot dry weight (F_5,29_ = 3.29, *P*=0.017). Compared to the control treatment, plants growing with one day of low DLI showed a significant decrease in dry weight of 13% (*P*=0.022). The other significant differences were between plants growing under T1 and T4 (*P*=0.047). In addition, we did not find a significant interaction between light treatment and cultivar (F_5,24_ = 0.33, *P*=0.88).

### 2.5 Shoot fresh weight

**Figure 7:**
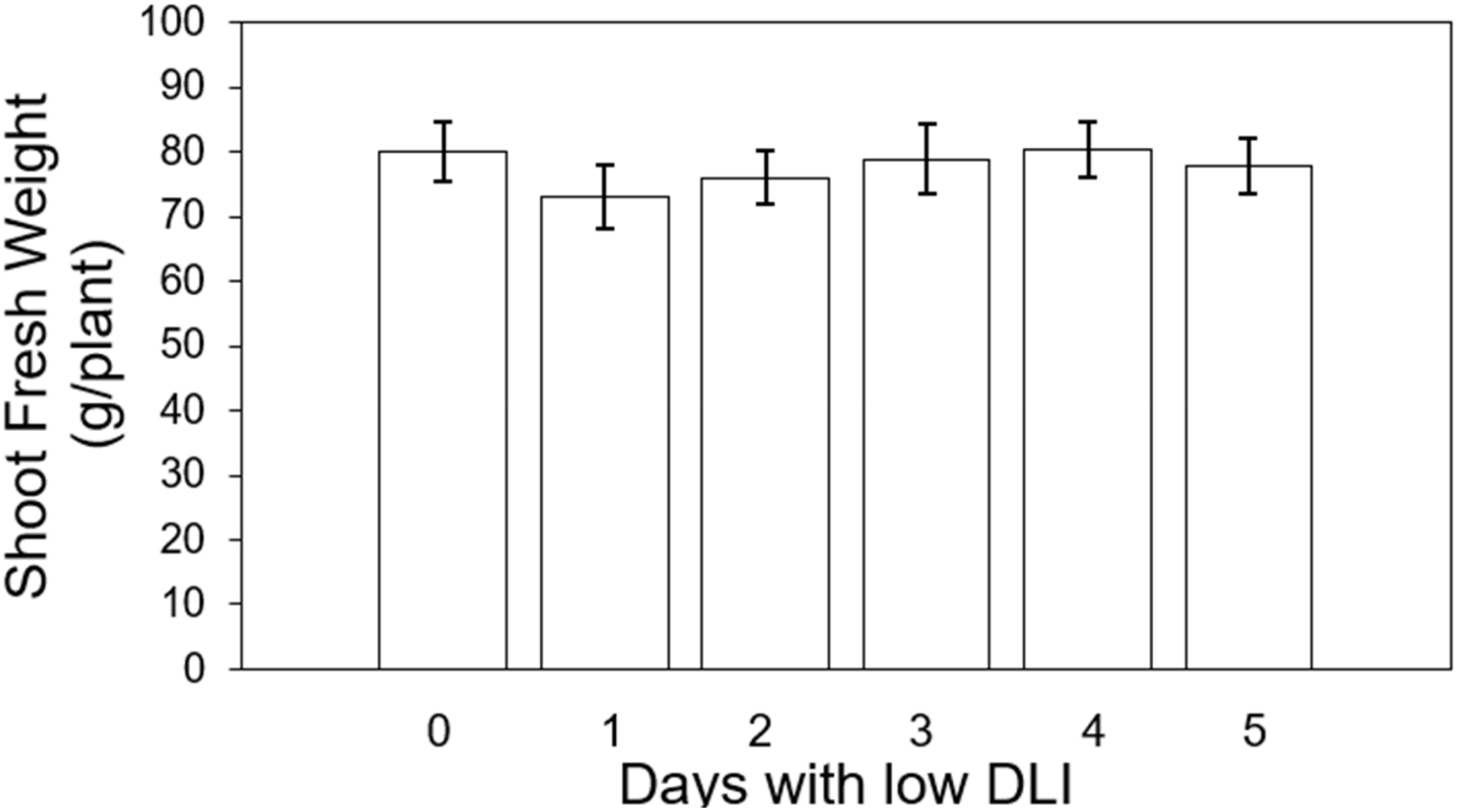
Final shoot fresh weight of ‘Rouxai’ and ‘Walmand’s dark green’ lettuce plants growing under different lighting treatments. Lighting treatments are described by the number of days with low daily light integral (DLI) after a day with high DLI. Error bars denote standard error (n=6). Absence of letters on top of error bars show no significant differences at α=0.05 from Tukey’s test.

On the other hand, we also assessed the final fresh weight of shoots across both cultivars. The values were the following from the treatment with less amount of days with low DLI (T0 days) to the one with more days with low DLI (T5): 80, 73, 76.07, 78.85, 80.42, and 77.95 g per plant respectively (**Figure 7**). Lighting treatments did not show significant differences (F_5,29_ = 1.48, *P*=0.22), and there was no significant interaction between cultivar type and lighting treatment (F_5,24_ = 0.49, *P*=0.77).

### 2.6 Light use efficiency

**Figure 8:**
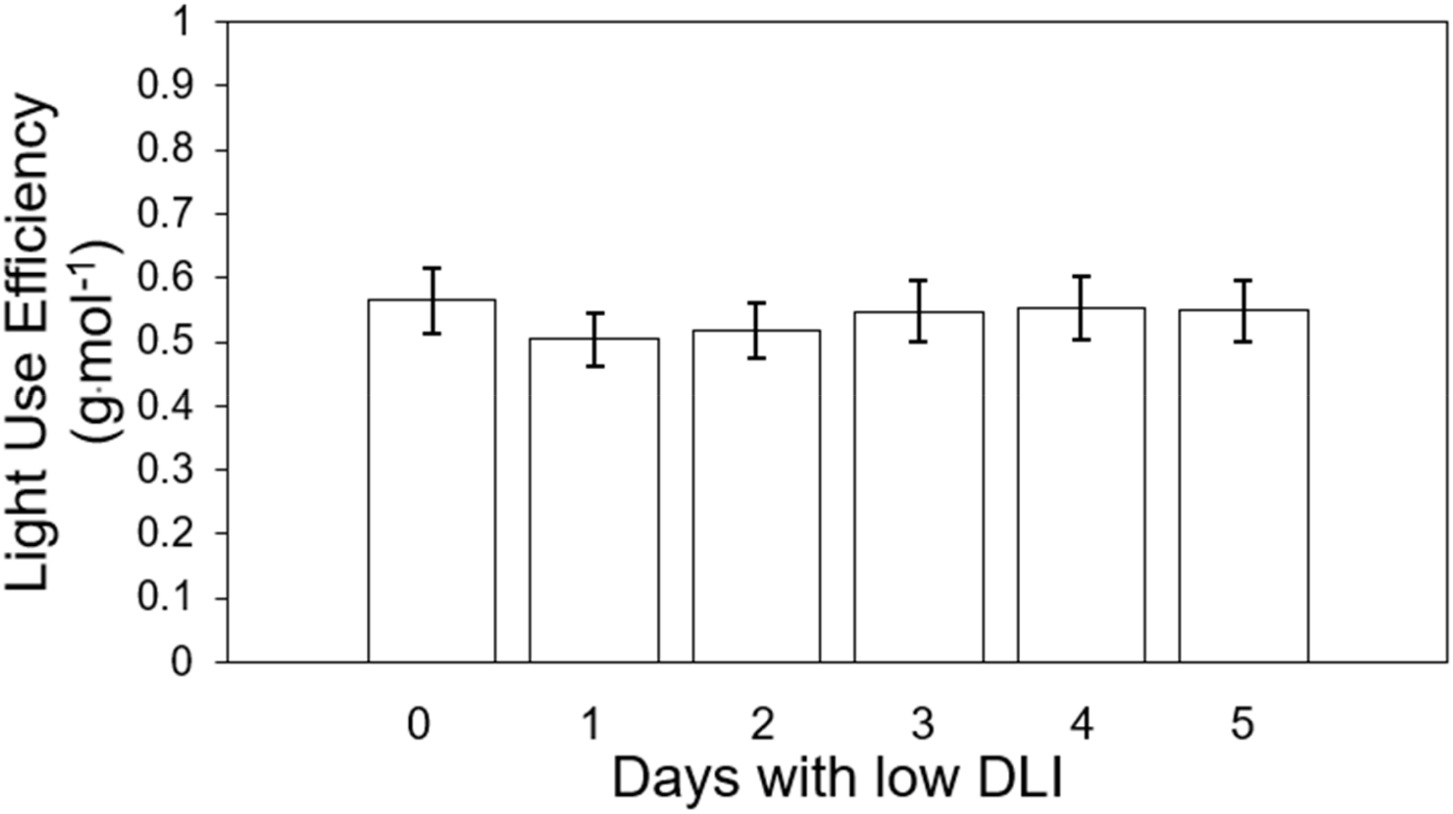
Light use efficiency of ‘Rouxai’ and ‘Walmand’s dark green’ lettuce plants growing under different lighting treatments. Lighting treatments are described by the number of days with low daily light integral (DLI) after a day with high DLI. Error bars denote standard error (n=6). Absence of letters on top of error bars show no significant differences at α=0.05 from Tukey’s test.

We calculated light use efficiency (LUE) for both cultivars for their whole growing cycle. The LUE for treatment from T0 to T5 were 0.56, 0.5, 0.51, 0.54, 0.55, and 0.54 g·mol^-1^, respectively (**Figure 8**). We did not find significant differences among lighting treatments on LUE (F_5,29_ = 1.92, *P*=0.121) or interaction between cultivar and treatment (F_5,24_ = 0.63, *P*=0.147). (F_5,24_ = 0.69, *P*=0.63).

### 2.7 Leaf number

**Figure 9:**
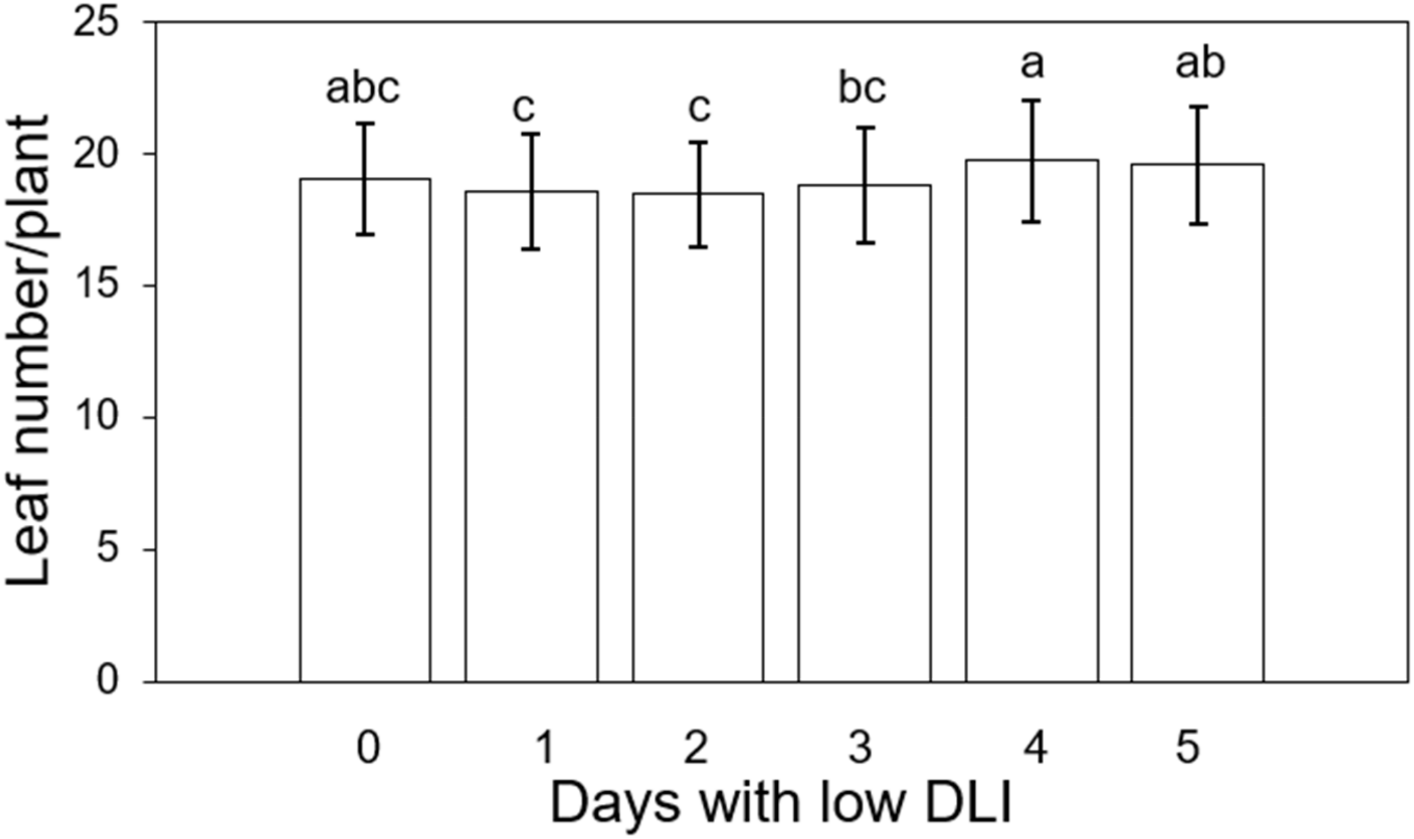
Final leaf number per plant of ‘Rouxai’ and ‘Walmand’s dark green’ lettuce plants growing under different lighting treatments. Lighting treatments are described by the number of days with low daily light integral (DLI) after a day with high DLI. Error bars denote standard error (n=6). Different letters on top of error bars show significant differences at α=0.05.

We counted the leaf number of three plants in the middle transversely of each tray, resulting in six plants counted (three per cultivar per treatment). The average leaf count per treatment, ranging from the lowest to the highest number of days with low DLI, was as follows: 19, 18.5, 18.44, 18.77, 19.72, and 19.55 leaves (**Figure 9**). The analysis of variance (ANOVA) test showed a significant difference in leaf number (F_5,29_ = 2.79, *P*=0.035). However, Tukey’s test did not show significant differences among the treatments. Additionally, we did not find an interaction between treatment and cultivar (F_5,24_ = 0.45, *P*=0.8). Then, we used the Fisher DLS test since Tukey’s test did not show the significant differences announced by the ANOVA. Significant differences were found between plants growing under T1 in comparison to T4 and T5, among plants growing under T2 in comparison to T4 and T5. Finally, there was a significant difference in leaf number between plants growing under T3 and T4. However, no significant differences existed between the treatment and the control treatment or plants growing with zero days with low DLI or no DLI fluctuations.

### 2.8 Energy requirement

**Figure 10:**
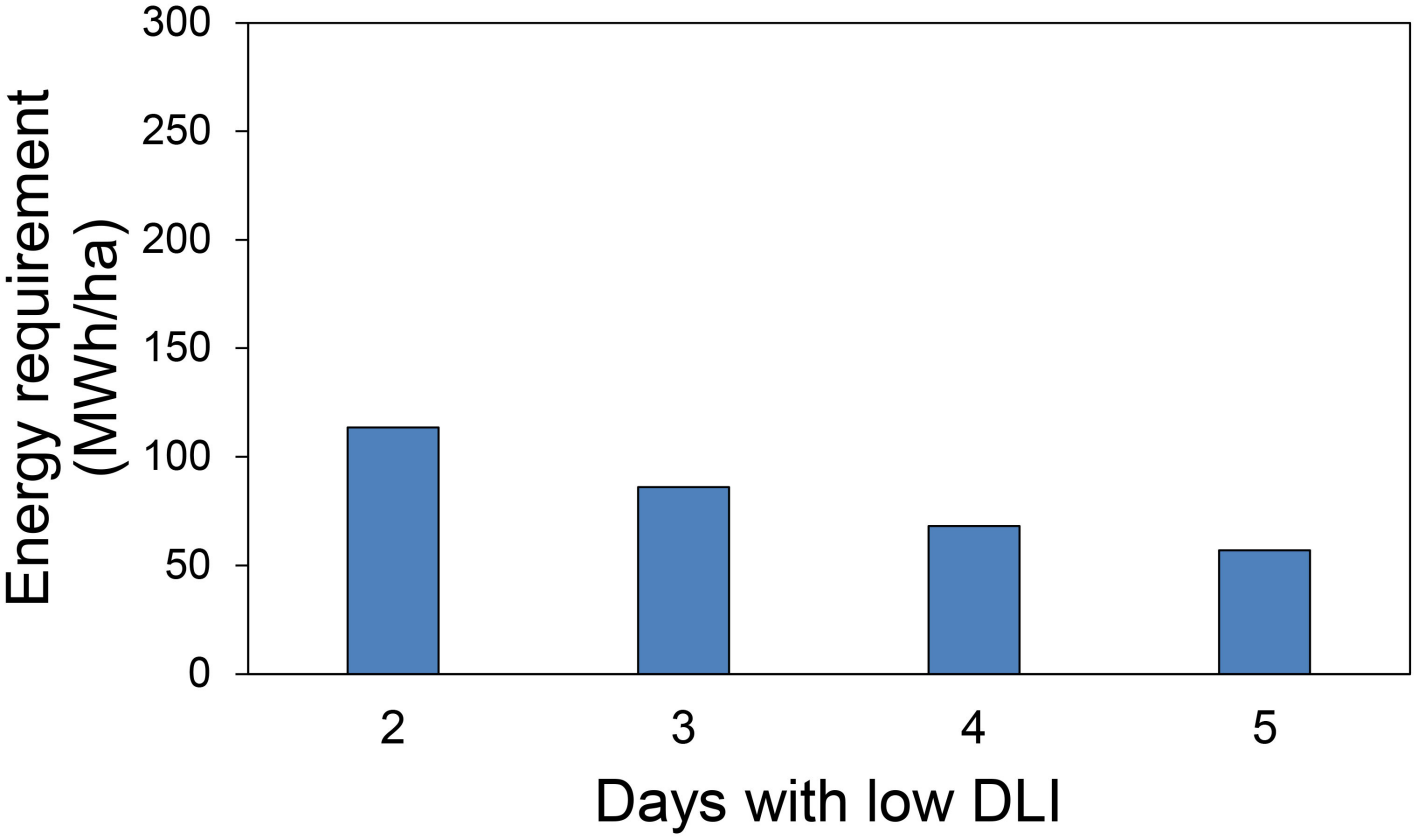
Energy requirement in 30 days used in supplemental lighting to provide extra daily light integral (DLI) to achieve a DLI of 15 mol·m^-2^·d^-1^ for days with low DLI for the first hypothetical case presented.

In the first hypothetical scenario about energy needs, we assessed the energy required to generate the additional DLI needed to reach the target DLI of 15 mol·m^-2^·d^-1^ on days with low DLI, considering the number of low DLI days in our treatments over a 30-day period. The energy requirements were as follows: 113.6 MWh/ha when the additional DLI was 3.75 (1 low DLI days), 86.1 MWh/ha for an extra DLI of 2.5 mol·m^-^²·d^-^¹ (2 low DLI days), 68 MWh/ha for 1.87 mol·m^-^²·d^-^¹ (3 low DLI days), and 56.9 MWh/ha for 1.5 mol·m^-^²·d^-^¹ (1 low DLI days) (**Figure 10**).

**Figure 11:**
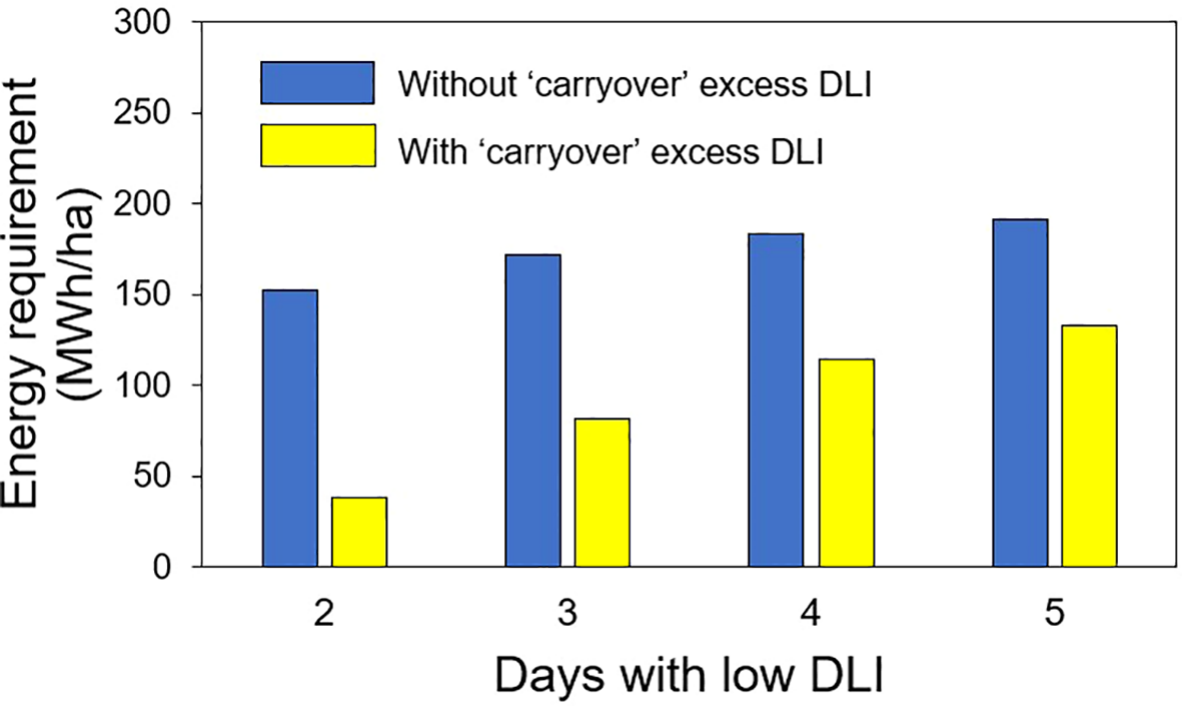
Energy requirement in 30 days used in supplemental lighting to provide extra daily light integral (DLI) to achieve a DLI of 15 mol·m^-2^·d^-1^ (without carryover) to obtain a lower DLI (with carryover depending on the number of days with low DLI) for days with low DLI for the second hypothetical case presented.

In the second hypothetical case of energy savings, we calculated the energy required to produce an additional DLI of 5 mol·m^-2^·d^-1^ (to reach the optimal 15 mol·m^-2^·d^-1^) on days with low DLI, assuming that sunlight provided a DLI of 10 mol·m^-2^;·d^-1^ over 30 days. The energy requirements were as follows: 152.7 MWh/ha for 2 days with low DLI after each day with high DLI, 171.8 MWh/ha for 3 days, 183.3 MWh/ha for 4 days, and 190.9 MWh/ha for 5 days of low DLI following a day with high DLI. Additionally, we assessed the energy needed to produce extra DLIs of 1.25, 2.5, 3.13, and 3.5 mol·m^-2^;·d^-1^ for 2, 3, 4, and 5 days of low DLI, respectively, following a day with high DLI. The resulting energy values were 38, 81.6, 114, and 133.3 MWh/ha for the additional DLI levels during the 30-day period (**Figure 11**).

## 3 Discussion

### 3.1 Plant growth rate

Plants growing under treatment1 day with high DLI followed only by 1 day with low DLI (T1) grew at a slower rate in comparison to plants growing with 4 (T4) and 5 days with low DLI (T5). We observed this while determining the time required for plants to reach 25%, 50%, and 75% coverage of a reference area. We assessed the plant growth rate using the PCS registered and calculated at various time points throughout the plant growth cycle. This means that when plants required more days to attain a specific coverage, they had a lower PCS, implying a slower growth rate. According to Klassen et al. (2003), plant growth is influenced by the quantity of light reaching the plant, which is directly tied to PCS and LUE (Legendre and van Iersel, 2021). Then, faster plant growth can be associated with a high PCS value. Plants with higher PCS intercept more light (Weaver and van Iersel, 2020), leading to more photosynthesis and biomass accumulation (Klassen et al., 2003). Said differences in biomass accumulation can be observed in **Figure 6**, what explains the differences in canopy sizes and plant growth rate in our study (**Figures 1A–C**).

**Figure 12:**
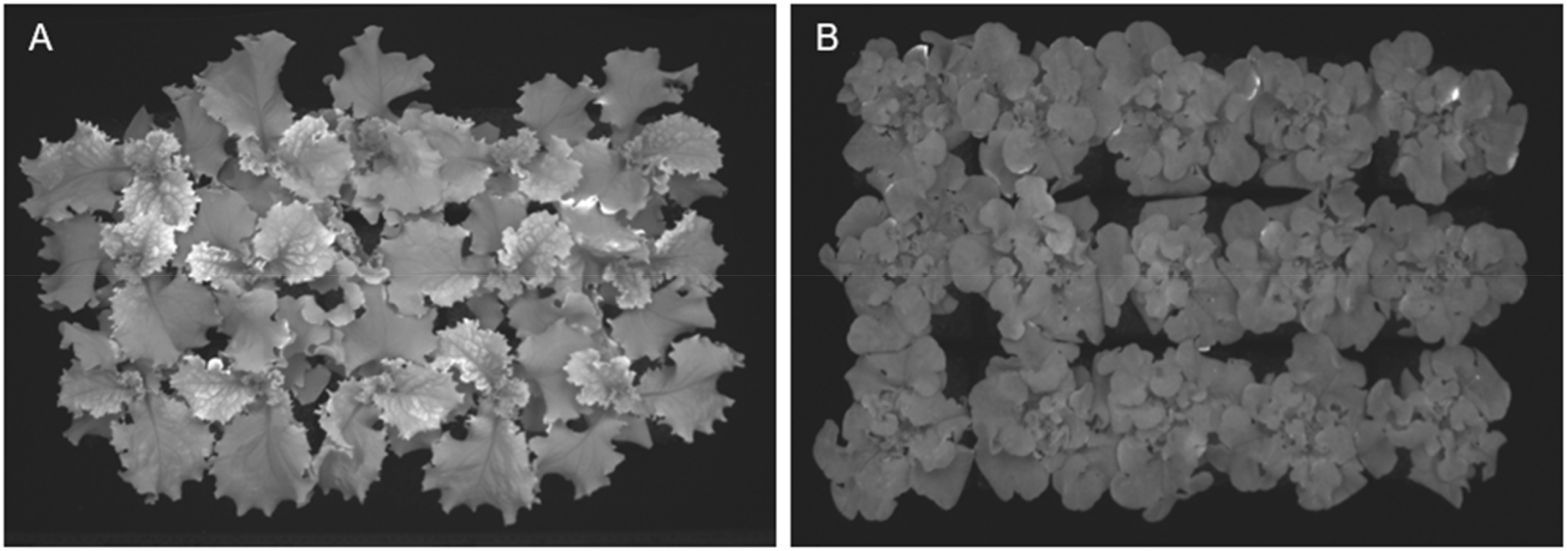
Picture of projected canopy size at 17 days after seeds were sowing shows how the canopy of individual plants overlaps with others. **(A)** ‘Walmand’s dark green’ plants. **(B)** ‘Rouxai’ plants.

As indicated by Jayalath and van Iersel (2021), LUE was one factor that plays a role in plant growth. LUE is a measure of the plant’s efficiency in producing biomass with light reaching its canopy (Legendre and van Iersel, 2021). Then, differences in LUE between the plants growing under distinct light treatments are expected to contribute to differences in PCS or growing rates. However, those significant differences disappear when calculating the number of days needed to cover 100% (**Figure 1D**) of the reference area, a consequence of the overlapping canopies of the plants (**Figure 12**).

### 3.2 Light use efficiency

LUE (**Figure 8**) followed a similar trend to fresh and dry weight despite the absence of significant differences. This means that plants, regardless of the treatment, had the same efficiency in producing biomass with the incident light provided (Jayalath and van Iersel, 2021). The intensity of light may influence variations in LUE. Elevated light levels lead to a greater closure of the reaction centers within the photosystem II (PSII). With an increased closure of these centers, a higher proportion of absorbed light by the PSII light-harvesting complex remains unused for electron transport in PSII (van Iersel, 2017). Excess of absorbed light might be dissipated in different ways (Bassi and Dall’Osto, 2021). Consequently, when the photosynthetic machinery does not utilize light, more photons are being redirected to other routes and not used by photosynthesis, resulting in lower LUE values. On the contrary, reduced light levels produced an inverse effect and a higher LUE. The treatments in our study were made up of a combination of days with low DLI or low light intensity and days with high DLI or high light intensity, which would mean different LUE depending on the day. However, said variations in light intensities across treatments did not significantly affect the final cumulative utilization of absorbed light for electron transport or dissipation, as evidenced by the absence of significant differences in LUE.

### 3.3 Shoot dry weight

Plants growing under 1 day with high DLI (22.53 mol·m^-2^·d^-1^) followed by 1 day with low DLI (7.53 mol·m^-2^·d^-1^) showed the lowest final dry shoot weight, meaning that the group of plants had a lower biomass accumulation (**Figure 6**). Dry weight increased when the number of days with low DLI increased, and at the same time, the DLI for those days increased (11.25, 12.5, 13.17, and 13.52 mol·m^-2^·d^-1^, respectively). As far as we know, most studies that look into DLI and biomass accumulation have grown plants under fixed DLI, even if that DLI is composed of a different combination of PPFD and photoperiod (Elkins and van Iersel, 2020). Jayalath et al. (2024) recently experimented with DLI changes only for two consecutive days. In our study, our treatments had variable DLIs for more than two following days, making it more challenging to compare results. Yan et al. (2019b) showed an increasing direct relationship between leaf dry weight and DLIs between 5.04 to 15.12 mol·m^-2^·d^-1^. Furthermore, Pennisi et al. (2020) reported the highest value in shoot dry weight for plants growing under a DLI of 14.4 mol·m^-2^·d^-1^ made of a photoperiod of 16 hours and a PPFD of 250 µmol·m^-2^·s^-1^. Plants growing under lower DLIs (5.8, 8.6, 11.5 mol·m^-2^·d^-1^) and a higher DLI (17.3 mol·m^-2^·d^-1^) showed significantly lower final biomass values for lettuce. This relationship of higher dry weight with high light levels has been also observed on dwarf tomato (*Lycopersicon esculentum*) ‘Micro-Tom’ (Ke et al., 2023), common purslane (*Portulaca oleracea*) (Kudirka et al., 2023), petunia (*Petunia × hybrida*) ‘Wave Blue’, geranium (*Pelargonium × hortorum*) ‘Pinto Premium Orange Bicolor’, and coleus (*Solenostemon scutellariodes*) ‘Wizard Golden’ (Park and Runkle, 2018).

In addition, differences in photosynthesis rates and canopy size may explain differences in final shoot dry weight in our study. Zhou et al. (2020) observed an increase in photosynthesis rates of lettuce plants with increasing PPFD levels, ranging from 0 to 350 μmol·m^-2^·s^-1^ (12-hour photoperiod of about DLI 15·mol·m^-2^·d^-1^), reaching a plateau phase at approximately 500 µmol·m^-2^·s^-1^. In our study, plants exposed to 1 day of low DLI (T1) received around 104 µmol m^-2^·s^-1^, while those subjected to 5 days of low DLI (T5) received approximately 187 µmol·m^-2^·s^-1^. This difference in incident light could signify variations in photosynthesis rates, and greater photosynthesis is associated with increased biomass accumulation (Klassen et al., 2003). However, all treatments were also exposed to a day with a high DLI of approximately 14.4 mol·m^-2^·d^-1^, achieved by subjecting plants to PPFD of around 312 µmol·m^-2^·s^-1^. Specifically, plants growing with just 1 day with low DLI (T1) had a DLI of 22 mol·m^-2^·d^-1^ during half of the growing cycle. This might suggest that these plants could have exhibited a higher photosynthetic rate during days with elevated PPFDs than other treatments (Zhou et al., 2020). However, it is crucial to note that, for the other half of the growing cycle, plants under T1 experienced lower DLI values (approximately 7.53 mol·m^-2^·d^-1^ and PPFD of 104 µmol·m^-2^·s^-1^). The potential reduction in the photosynthetic rate at these lower light levels could account for a decrease in the final biomass. Zhou et al. (2020) showed reduced photosynthetic values when light intensity was low. Additionally, Pennisi et al. (2020) showed lower values of biomass accumulation for plants growing under a DLI of 8.6 mol·m^-2^·d^-1^ and PPFD of 150 µmol·m^-2^·s^-1^.

When the number of days with low DLI increased, the minimum DLI also increased. Plants growing for 4 and 5 days with low DLI had an average DLI of 13.17 and 13.52 mol·m^-2^·d^-1^, respectively. Hence, those plants were growing most of the days under a DLI that was close to the control treatment (14.88 mol·m^-2^·d^-1^), which is close to the ideal DLI for lettuce between 12 and 16mol·m^-2^·d^-1^ depending on the cultivar (Zhang et al., 2018; Yan et al., 2019a; Kelly et al., 2020; Pennisi et al., 2020; Modarelli et al., 2022). Consequently, the photosynthetic rates, LUE, and biomass accumulation of plants growing under T4 and T5 are higher (in comparison to T1) and similar to the control treatment. Jayalath et al. (2024) reported a decrease in final dry weight when the DLI fluctuation from one day to the next is above 15 mol·m^-2^·d^-1^. They also explained this partly due to a lower canopy rate expansion that decreased the incident light available for photosynthesis. In our study, this lower expansion rate on the canopy can be seen in the number of days plants needed to reach different sizes (**Figure 1**).

These results corroborate the hypothesis the carryover effect exists, wherein higher photosynthesis rates during days with high DLI might compensate for the lower photosynthetic rates observed on days with lower DLI. They also indicate that plants experiencing more days with low DLI maintain similar photosynthetic rates because these lower DLI values are closely aligned with the optimal conditions required for lettuce growth.

### 3.4 Shoot fresh weight

Despite the significant differences observed in shoot dry weight results among the different lighting treatments, these differences were not found when measuring the final shoot fresh weight (**Figure 7**). However, the consistent trend of increasing dry weight when the number of days with low DLI increased was also observed in the fresh weight. Changes in significant differences between shoot dry and fresh weight have been reported before. Khwankaew et al. (2018) reported mismatched significant differences between shoot dry and fresh weight on *Ipomoea aquatica* plants growing under LED light with different spectrum combinations. Fresh weight should be assessed promptly after sampling, or plant material should be stored in hermetically sealed recipients, as fresh plant material tends to lose water rapidly (Turner, 1981). Then, if fresh weight is not taken consistently across all the samples, some variation might be induced. In our study, we measured this parameter right after cutting the shoot from the root system. Therefore, we did not expect any variability in fresh weight induced by the sampling process. A possible reason for the differences in dry weight not being reflected in the final fresh weight could be related to the water status of the plants and their water retention capacity. For instance, plants growing under our control conditions showed a fresh weight of around 80 g per plant and a dry weight of approximately 4 g per plant. We can see that the final dry weight represented only 5% of the total fresh weight of the plants. This means that the significant differences observed in dry weight are lost due to the amount of water inside the leaf. Fresh weights have been reported in other studies to be more variable for showing differences among treatments compared to dry weights (Bashan and De-bashan, 2005; Huang et al., 2017).

### 3.5 Leaf area and specific leaf area

No significant differences were found in final leaf area or SLA in our study (**Figures 2**, **3**). Pennisi et al. (2020) observed an increase in leaf area for lettuce and basil with higher DLI. In contrast, the SLA for both species decreased as DLI increased. This reduction in SLA was attributed to the denser arrangement of mesophyll cells and the development of thicker and larger leaves. Similarly, Carotti et al. (2021) found that SLA decreased when lettuce plants grew under higher light intensities. However, these results were obtained for plants growing under constant DLI or light intensities. Bhuiyan and van Iersel (2021) grew lettuce plants under variable PPFD levels every 15 minutes (400/0, 360/40, 320/80, 280/120, 240/160, and 200/200 μmol·m^-2^·s^-1^), and observed that leaf area decreased when plants were subjected to high PPFD fluctuations but increased when the PPFD fluctuations were minimized between light levels. In contrast, SLA was higher under high PPFD fluctuations, whereas it was lower when the fluctuations were smaller at their respective light levels. They argue that extreme fluctuations in light prevent plants from reaching a steady state of photosynthesis. Consequently, this leads to reduced carbon gain by leaves, resulting in decreased dry weight and increased SLA. In our study, the DLI and PPFD fluctuations occurred over an extended period, suggesting that plants could reach a steady state of photosynthesis. This likely contributed to the production of similar biomass across treatments, resulting in comparable SLA values. Given the similar biomass production, we expected similar leaf area values regardless of our lighting treatments.

For plants growing under DLI fluctuations that happened during extended periods, specifically focusing on day-to-day variations, Jayalath et al. (2024) found that DLI fluctuations above 15 mol·m^-2^·d^-1^ significantly reduced leaf area. Lower PCS and LUE may explain these differences. A larger PCS captures more light, and higher LUE transforms that light more efficiently into biomass. While our study also observed differences in PCS initially, we found that PCSs became similar across all treatments once canopies started to overlap. This suggests that plants reached similar leaf area values with similar PCS and LUE. Nonetheless, our treatment involving a single day with low DLI exhibited a DLI fluctuation from one day to the next of approximately 15 mol·m^-2^·d^-1^, consistent with findings reported for lettuce (Jayalath et al., 2024). Despite this fluctuation, we did not observe significant differences in leaf area under the same lighting treatment.

### 3.6 Leaf number

Even when we found significant differences in leaf number among the treatments involving a DLI fluctuation, none showed a significant difference compared to the control treatment (**Figure 9**). Changes in leaf number depending on different light levels have been reported before. Kang et al. (2013) found a higher leaf number for lettuce plants growing under high PPFD (290 μmol·m^-2^·s^-1^) levels when their photoperiod was 6/2 (light/dark) in 3 cycles per day. On the other hand, the least number of leaves was found when the plant grew at a low PPFD (200 μmol·m^-2^·s^-1^) with a photoperiod of 18/6 (light/dark). Also, Zervoudakis et al. (2012) found a significant reduction of leaf number in common sage (*Salvia officinalis* L.) when they grew under 25% ambient light compared to plants growing under full ambient light. These results suggest that plants growing under lower light levels generally produce fewer leaves. In our study, even plants that grew under different PPFD levels depending on the number of days with low DLI; they had, on average, the same light levels when taking into account the days with DLI and the days with low DLI. This might explain the lack of differences in leaf number compared to the control treatment (T0).

### 3.7 Relative pigment content

#### 3.7.1 Anthocyanins

We did not see any differences in leaf anthocyanin content on ‘Rouxai’ plants regardless of the lighting treatment those plants were growing under (**Figure 4**). Hwang et al. (2023) demonstrated that elevating light intensity and extending the photoperiod increased anthocyanin content in *Brassica juncea* cultivated in a plant factory. The highest concentrations of anthocyanins were observed in plants exposed to 300 µmol·m^-2^·s^-1^ for 18 hours daily. Likewise, Jones-Baumgardt et al. (2020) reported a higher concentration of anthocyanins in arugula (*Eruca vesicaria* subsp. *sativa* (Mill.) Thell.), cabbage (*Brassica oleracea* L.), kale (*Brassica napus* L. subsp. *napus* var. *pabularia* (DC.) Alef.), and mustard (*Brassica juncea* (L.) Czern) plants when cultivated under 600 µmol·m^-2^·s^-1^, as opposed to those grown under 100 µmol m^-2^·s^-1^. Similar results were found on arugula (Veremeichik et al., 2023), pak-choi (*Brassica campestris* ssp. Chinensis Makino) (Zhu et al., 2017), sweet basil (*Ocimum basilicum*) ‘Opal’ and lettuce (*Lactuca sativa*) ‘Nikolaj’ (Sutulienė et al., 2022), lettuce ‘Outredgeous’ (Massa et al., 2015) and red mustard (Hwang et al., 2023). One potential role of anthocyanins in plants is safeguarding the photosynthetic machinery against high light intensities (Landi et al., 2015). Anthocyanins are believed to play a role in partially mitigating the impacts of de-epoxidation of violaxanthin within the photo-protective xanthophyll cycle (Cavender-Bares et al., 1999; Landi et al., 2015; Logan et al., 2015).

The plants in our study experienced comparable high PPFD levels during the high DLI day. Those subjected to 1 to 5 days with low DLI received approximately 312 µmol·m^-2^·s^-1^ intermittently throughout their growth cycle, potentially leading to a similar production of anthocyanins. However, under the control treatments, plants did not have days with low DLI or PPFD levels. Then, plants probably still needed protection for their photosynthetic machinery.

Temperature changes have been linked to variations in anthocyanin levels. According to Gazula et al. (2005) ‘Lollo Rosso’ lettuce exhibited a notable increase in anthocyanin content when grown at lower temperatures than plants cultivated under higher temperatures. Similar response was observed on Chinese cabbage (*Brassica rapa* L.) (He et al., 2020), strawberry (*Fragaria × ananassa* Duch. cv. Toyonoka) (Zhang et al., 2018), Japanese parsley (*Oenanthe stolonifera*, DC.) (Hasegawa et al., 2001), and grape (*Vitis labrusca* L. × *Vitis vinifera* L.) (Gao-Takai et al., 2019). Lower temperatures are believed to lead to elevated transcript levels for enzymes like phenylalanine ammonia-lyase and chalcone isomerase, which play a role in anthocyanin biosynthesis (Dela et al., 2003). Plants in our investigation experienced uniform temperature conditions, which could partly account for the absence of significant differences in anthocyanin content.

#### 3.7.2 Chlorophyll

Similar to anthocyanins in ‘Rouxai’ plants, we did not find significant differences in chlorophyll content for either ‘Rouxai’ or ‘Walmand’s dark green’ plants, regardless of the lighting treatment (**Figure 5**). Chlorophyll content per unit area indicates the plant’s photosynthetic capacity could have been influenced by environmental factors (Palta, 1990). For instance, chlorophyll may degrade in excess light (De Carvalho Gonçalves et al., 2005) due to photo-oxidation (Kramer and Kozlowski, 1979), while under low light conditions, the content of this pigment might increase (Czeczuga, 1987). Zervoudakis et al. (2012) reported an increase of chlorophyll content on *Salvia officinalis* when growing under low light conditions. Similarly happened to sweet pepper (*Capsicum annuum* L.) (Sui et al., 2012). In our study, plants were subjected to varying light levels (high and low) across different days without any differences in chlorophyll content. This could indicate that the plants might have reached a balance in their suitable chlorophyll content for both conditions. Conversely, two rice (*Oryza sativa*) phenotypes growing under different lighting conditions, 600 and 1200 µmol·m^-2^·s^-1^, did not show significant differences in chlorophyll content despite the light intensity (Zhao et al., 2017). This suggests that variations in chlorophyll content in response to light conditions might vary among different species.

### 3.8 Energy requirement

DLI fluctuations only from one day to another or “carryover” effect has been reported to potentially reduce energy cost related to supplemental lighting in green gouses set up (Jayalath et al., 2024). In this study, we tested how plants behave when said DLI fluctuations happen during multiple days. We proposed two hypothetical cases to assess the energy requirements for supplemental light when fluctuating DLI levels during various days. For the first case, we assumed that plants would be subjected to DLI of 11.25, 12.5, 13.13, and 13.5 mol·m^-2^·d^-1^ (provided by sunlight) during 2, 3, 4, and 5 days after a day with high DLI (22.5 mol·m^-2^·d^-1^) respectively. Under the conditions of our study, said plants did not require a DLI of 15 mol·m^-2^·d^-1^ after a day with high DLI to not show significant differences in plant growth compared to plants growing consistently under the optimal DLI of 15 mol·m^-2^·d^-1^. It has been showed that plants could be allowed to get a high DLI on sunny days, and this could compensate for a day with a low DLI immediately after, and this concept was called the carryover effect (Jayalath et al., 2024). Then, if we experience one day with a high daily light integral (DLI) of 22.5 mol·m^-2^·d^-1^, followed by subsequent days with lower DLIs of 11.25, 12.5, 13.13, or 13.5 mol·m^-2^·d^-1^ over the next 2, 3, 4, or 5 days respectively, all under natural light conditions (as described in our first hypothetical case), the use of energy for supplemental lighting (**Figure 10**) to achieve an optimal DLI of 15 mol·m^-2^·d^-1^ may not be necessary. This is due to the carryover effect from the initial high DLI day.

In our second hypothetical case, we calculated the energy requirements assuming that days with a high DLI of 22.5 mol·m^-^²·d^-^¹ (from sunlight) would be followed by periods of 2, 3, 4, and 5 days with low DLI. Additionally, we assumed that these low DLI days would receive only 10 mol·m^-^²·d^-^¹ (also from sunlight). We estimated the energy needed to supplement an extra 5 mol·m^-2^·d^-1^ to achieve the optimal 15 mol·m^-^²·d^-^¹, as well as the energy required to generate additional DLI to reach 11.25, 12.5, 13.13, and 13.5 mol·m^-^²·d^-^¹ (depending on the number of consecutive days with low DLI) to take advantage of the ‘carryover’ effect. In our study, the contrast in energy requirements between achieving the optimal DLI and the energy to use the ‘carryover’ effect could indicate potential energy savings.

## 4 Materials and methods

### 4.1 Experimental set up and treatments

This research was conducted in a walk-in growth chamber (vertical farm) at the University of Georgia (College of Agriculture and Environmental Sciences, Department of Horticulture, Horticultural Physiology Laboratory), in Athens, GA, USA. The environmental conditions during the experiment, without distinguishing between light and dark periods, were: (mean ± standard deviation): temperature 24.35 ± 0.673°C, relative humidity 65.22% ± 7.56%, CO_2_ concentration 847.64 ± 43.52 mg/L, and vapor pressure deficit 1.0201 ± 0.235 kPa.

Inside the growth chamber, there were three metal racks (2.4 m long × 0.6 m wide × 2.2 m high), each serving as a separate replication. Each rack had three horizontal shelves, and each shelf was divided into two equal parts vertically, resulting in six growing spaces per rack and 18 growing spaces in total, each with dimensions of 1.2 m long × 0.6 m wide × 0.6 m high. Each growing space was equipped with two LED fixtures (SPYDRx Plus with PhysioSpec indoor spectrum; Fluence Bioengineering, Austin, TX, USA) (**Supplementary Figure 1**). Furthermore, four small fans (AD0412HB-C50; ADDA, Orange, CA, USA), evenly distributed, were positioned on the sides of each growing space to ensure proper lateral airflow.

We tested six lighting treatments randomly assigned to the growing spaces. These lighting treatments were controlled by a datalogger (CR6; Campbell Scientific, Logan, UT, USA) and six dimmable drivers (4009715; Intertek/Fluence, Arlington, VA, USA), with each driver responsible for controlling three growing spaces that shared the same lighting treatment, one space per rack or replication.

**Figure 13:**
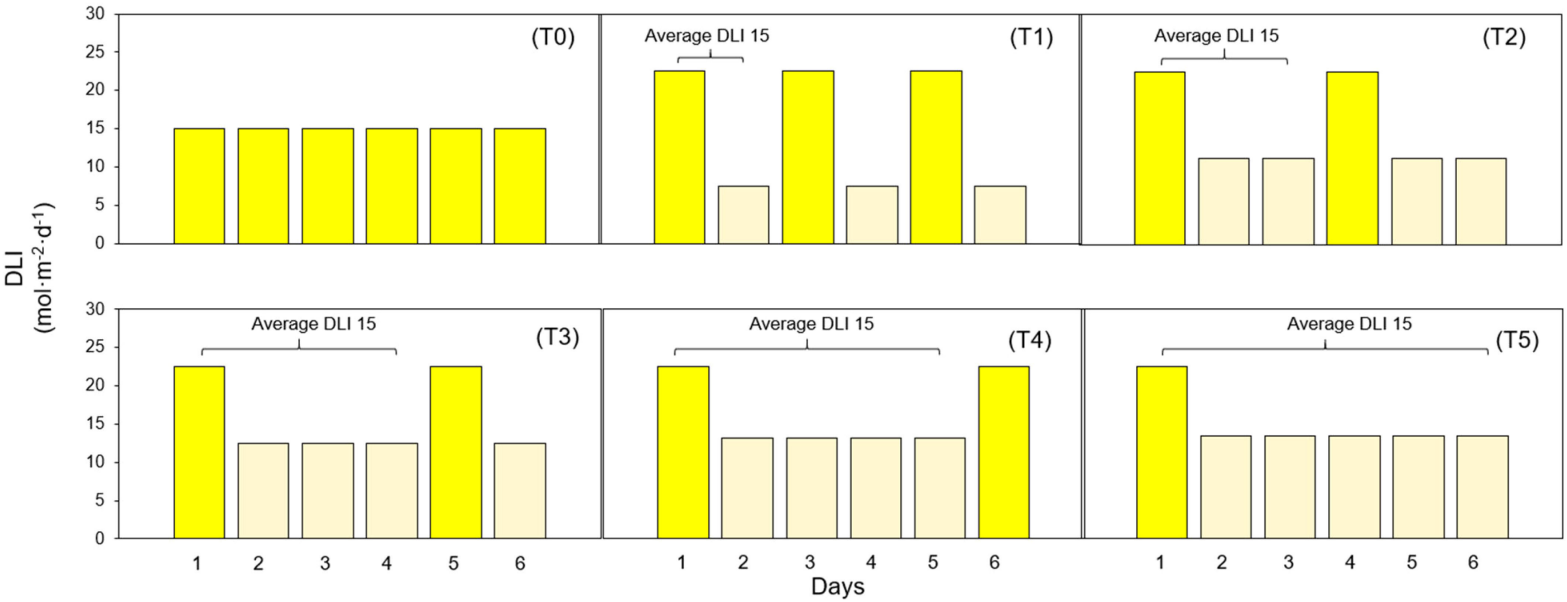
Diagram showing different lighting treatments. (T0) Treatment with zero days with low DLI. (T1) Treatment with one day with high DLI followed by one day with low DLI. (T2) Treatment with one day with high DLI followed by two days with low DLI. (T3) Treatment with one day with high DLI followed by three days with low DLI. (T4) Treatment with one day with high DLI followed by four days with low DLI. (T5) Treatment with one day with high DLI followed by five days with low DLI. The average DLI for each combination of days with high DLI and low DLI is 15 mol·m^-2^·d^-1^.

PPFD levels were assessed in the middle of every cultivation area using a quantum sensor (MQ-500; Apogee Instruments, Logan, UT, USA). Each treatment consisted of two DLI levels, called high and low, with a photoperiod of 20 hours. Plants were exposed to one day under high DLI, followed by varying numbers of days under low DLI, denoted as T0 (Control), T1, T2, T3, T4, and T5, indicating the respective number of days with low DLI in each treatment (**Figure 13**). DLI and PPFD for each treatment are shown in **Table 1**.

**Table 1:** Photosynthetic Photon Flux Density (PPFD) levels and corresponding daily light integral (DLI).

Treatment	High DLI	Low DLI	Average DLI	Days with low DLI	Photo-period	High PPFD	Low PPFD
	—— (mol·m^-2^·d^-1^) ——				(h)	— (µmol·m^-2^·s^-1^) —	
0 (Control)	14.88 ± 0.27	14.88 ± 0.27	14.88	–	20	206.66 ± 3.78	206.66 ± 3.78
1	22.53 ± 0.21	7.53 ± 0.18	15.03	1	20	313.00 ± 3	104.66 ± 2.51
2	22.48 ± 0.18	11.25 ± 0.18	15.00	2	20	312.20 ± 2.51	156.33 ± 2.51
3	22.44 ± 0.34	12.50 ± 0.2	14.98	3	20	311.66 ± 4.75	173.66 ± 3.78
4	22.51 ± 0.21	13.17 ± 0.19	15.04	4	20	312.66 ± 3.05	183.00 ± 2.64
5	22.48 ± 0.1	13.51 ± 0.14	15.01	5	20	312.33 ± 1.52	187.66 ± 2.08

High DLI, low DLI, high PPFD, and low PPFD are averages of three lighting fixtures with ± SD. Average DLI is the average of one day with high DLI and a different number of days with low DLI, depending on the treatment.

### 4.2 Plant material

Ten-cm square plastic pots were filled with soilless substrate (Metro-Mix^®^ 830; SunGro Horticulture, Agawam, MA, USA) up to about 1 cm below the top rim. Three pelleted ‘Waldmann’s dark green’ or ‘Rouxai’ (Johnny’s Selected Seeds, Winslow, ME, USA) seeds were planted in each pot. The substrate was then covered with calcined clay or metakaolin (Turface MVP; Turface Athletics, Buffalo Grove, IL, USA) to avoid algae growth affecting PCS measurement. These pots were organized in trays, with fifteen pots arranged in a 5×3 configuration and placed in the designated growing spaces. Once the seeds germinated, a thinning process was carried out to keep only one seedling per pot. The plants received irrigation and nutrients through an ebb-and-flow subirrigation system, which delivered a 15N–2.2P–12.45K nutrient solution containing 100 mg·L^-1^ of nitrogen using a water-soluble fertilizer (15-5-15 Ca-Mg Professional LX; J.R. Peters, Allentown, PA, USA).

### 4.3 Data collection and calculations

#### 4.3.1 Projected canopy size and plant growth rates

Canopy photos of 15 plants from each tray were captured initially 7 days following seed sowing and then twice a week, employing the setup detailed in Jayalath and van Iersel (2021). These images were analyzed using a custom Python script to calculate the PCS at the time said images were taken. The PCS data was then plotted against the number of days after sowing (DAS), and a sigmoidal curve of the form PCS = a/[1 + e^−(DAS−x0)/b^] was applied to fit the data (SigmaPlot 11.0; Systat Software, San Jose, CA, USA). From the regression equations, we estimated the PCS for each day during the growth cycle, spanning from day 1 to day 30 for ‘Wadmands’ dark green’ and to day 35 for ‘Rouxai’ (30 and 35 DAS were the harvest point respectively). Additionally, we calculated the number of days required for the crops to achieve specific sizes, such as 25%, 50%, 75%, and 100% coverage of the trays holding the plants (equivalent to 0.15 m^2^), based on the estimated PCS for each day throughout the growth cycle.

#### 4.3.2 Incident light and light use efficiency

To determine the daily incident light received by each group of plant’ canopies on each day of the growth cycle, we multiplied the daily PCS by the DLI for each light treatment, as expressed by the formula: Incident Daily Light Integral (mol·d^-1^) = PCS (mm^2^) × DLI (mol·m^-2^·d^-1^). Using these values, we calculated the cumulative incident light on the canopy over the entire growth cycle. Subsequently, the cumulative incident light was divided by the final dry weight of the shoot to calculate LUE.

#### 4.3.3 Leaf area and leaf specific area

We measured the leaf area of three plants per cultivar per growing space using a leaf area meter (LI-3100; LI-COR Biosciences, Lincoln, NE, USA) at harvesting day. The chosen plants were located transversally in the middle of each tray containing the pots. Then, we calculated SLA as the ratio between dry weight and leaf area.

#### 4.3.4 Pigment content

At harvesting (30 DAS for ‘Wadmands’ dark green’ and 35 DAS for ‘Rouxai), we assessed the relative pigment content of chlorophyll and anthocyanins. The measurement involved randomly selecting 10 plants (sub-samples) per growing space per cultivar for pigment content evaluation. Meassurements were taken on uppermost fully expanded leaves. The respective devices averaged the final value from each set of 10 measurements. Chlorophyll content for both cultivars was measured using a chlorophyll content meter (MC-100; Apogee Instruments, Logan, UT, USA) measuring the ratio of optical transmission at 931 to 653 nm. Anthocyanin measurement for ‘Rouxai’ was taken with an anthocyanin content meter (ACM 200 plus; Opti-sciences, Hudson, NH, USA) measuring the optical absorbancy at 530 and 931 nm.

#### 4.3.5 Fresh and dry weight

Following the harvest of plant shoots in each cultivation area at 30 DAS for ‘Wadmands’ dark green’ and 35 DAS for ‘Rouxai’, all of the plants (15 per cultivar per treatment) were weighed to obtaintheir fresh weight, and subsequently, dried in an oven at 80°C for 72 hours for final dry weight determination. We computed LUE as the ratio of shoot biomass to the total incident light.

#### 4.3.6 Energy requirement assessment

Finally, using two hypothetical scenarios, we assessed the theoretical energy requirements of implementing the lighting strategy of reducing the target DLI for multiple days after a sunny day or day with high DLI. In the first one, we assumed that DLI was obtained only from natural light (sunlight). Here the DLI were the same as the ones provided in the treatments of this study. Days with high DLI had a DLI of 22.5 mol·m^-2^·d^-1^, and the days with low DLI had a DLI of 11.25, 12.5, 13.13, and 13.5 mol·m^-2^·d^-1^ for treatments T2 to T5 respectively. Since 15 mol·m^-2^·d^-1^ is the optimal DLI reported to grow lettuce, we calculated the DLI needed to provide during the days with low DLI to achieve said ideal number. In this case, for every day with low DLI, T2 would need an extra 3.75 mol·m^-2^·d^-1^, T3 would need an extra 2.5 mol·m^-2^·d^-1^, T4 would need an extra 1.87 mol·m^-2^·d^-1^, and T5 would need an extra 1.5 mol·m^-2^·d^-1^ (**Supplementary Table 1**). To calculate the energy needed to produce the extra DLI, we followed the steps explained by Mattson (2017) and adopted the same assumptions. However, for this study, we used a light output of 1100 µmol·s^-1^, and an energy requirement of 600 watts for this light source and area to cover one hectare during 30 days. The count of days with low DLI over 30 days, as per our lighting regimes, was as follows: 20 days of low DLI when having two days of low DLI after a day with high DLI, 22.5 days when having three days with low DLI after a day with high DLI, 24 days if there were four days with low DLI after a day with high DLI, and 25 days when having five days with low DLI after a day with high DLI.

In the second hypothetical case, days with high DLI also experienced a DLI of 22.5 mol·m^-2^·d^-1^ naturally. However, the days with low DLI only received 10 mol·m^-2^·d^-1^ from the sun. We chose a DLI of 10 mol·m²·d-¹ because this represents the average daily amount of light received in a northern U.S. state such as Washington, during December, January, and February when light availability is lower (Faust and Logan, 2018). The average DLI for these months in this region typically ranges between 5 and 15 mol·m^-2^·d^-1^. For simplicity, we used the midpoint value of 10 mol·m^-2^·d^-1^ in our calculations. Then, the extra DLI would be provided with supplemental light either to obtain a DLI of 15 mol·m^-2^·d^-1^ to achieve the optimal literature value, or to achieve DLI of 11.25, 12.5, 13.13, and 13.5 mol·m^-2^·d^-1^ (depending on the number of days with low DLI) to use the ‘carryover’ effect (**Supplementary Table 2**). The energy required to produce the extra DLI to get the optimal value and to take advantage of the carryover was calculated as mentioned before.

All previous calculations were based on hypothetical DLI values provided by natural light (sunlight) and the hypothetical DLI values for each specific case using artificial lighting. The calculations considered only the DLI and did not account for the length of the day or night. It was assumed that the hypothetical DLIs from natural and supplemental light were provided over a period of 20 hours per day to provide a period of darkness even though lettuce does not respond to photoperiod.

### 4.4 Data analysis and experimental design

The experimental setup followed a randomized split-block design with three blocks and six lighting treatments. Each experimental unit consisted of 15 plants. We used ANOVA by using an statistical software (R version 4.1.2; R Project for Statistical Computing, Vienna, Austria) to compare differences in the number of days plants need to reach specific sizes, differences in shoot-dry weight, pigment content, leaf area, relative leaf area, dry and fresh weights, leaf number and light use efficiency.

## 5 Conclusions

After comparing plant responses under the lighting conditions tested in our study, we found that plants can tolerate multiple days with suboptimal DLI if they have been preceded by a day with higher-than-optimal DLI. This study suggests that plants can use the high DLI from one day to compensate for subsequent days with lower DLI. This finding implies that growers might not always need to achieve a specific DLI through supplemental lighting if similar lighting patterns are provided by sunlight. Consequently, this could reduce the need for supplemental lighting and result in economic benefits. However, the precise energy savings may vary depending on factors such as geographical location, weather conditions, and supplemental lighting systems. Additionally, determining the optimal DLI levels and acceptable DLI fluctuations for different crops is necessary if the findings of this study are to be applied more broadly.

## Data Availability

The raw data supporting the conclusions of this article will be made available by the authors, without undue reservation.
