# Supplementary material for: Lowering the target daily light integrals following days with excessive lighting can reduce lettuce production costs
Source: Front Plant Sci. 2024 Dec 10;15:1467443. doi: 10.3389/fpls.2024.1467443 (PMC11667103; doi:10.3389/fpls.2024.1467443)
Supplement: Supplementary file 1 [file DataSheet1.docx]

Supplementary Material

# Supplementary Figures and Tables


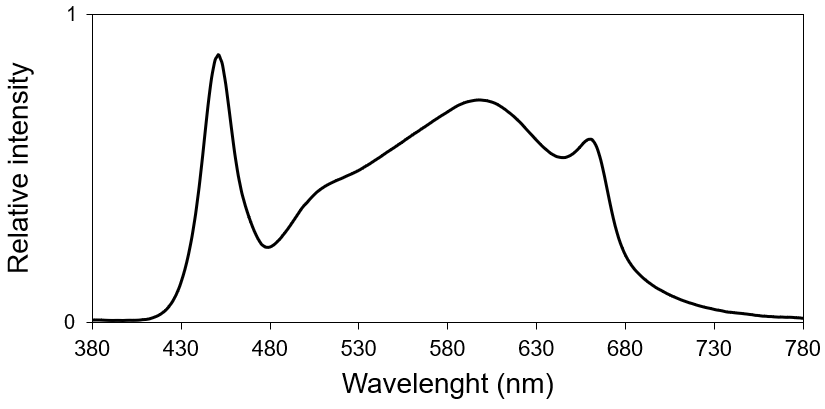


Supplemental figure 1. The electromagnetic spectrum emitted by LED fixtures (SPYDRx Plus with PhysioSpec indoor spectrum, Fluence Bioengineering, Austin, TX, USA)

Supplemental table 1. Lighting regime for the first hypothetical case to assess energy requirement depending on the number of days with low DLI after a day with high DLI. Lines in yellow indicate the day with high DLI, and lines in white indicate days with low DLI. For each combination of days with low DLI after a day with high DLI (lighting regime), we indicate the DLI received from the sun, the extra DLI to be achieved, the DLI reported in the literature for optimal plant growth, and the extra DLI to complete a DLI to take advantage of the ‘carryover’ effect.

|  | Lighting regime | | | | | | | | | | | |
| --- | --- | --- | --- | --- | --- | --- | --- | --- | --- | --- | --- | --- |
|  | 2 days with low DLI | | | 3 days with low DLI | | | 4 days with low DLI | | | 5 days with low DLI | | |
| Days | DLI from sun | Extra DLI to complete literature value | Extra DLI to complete carryover effect | DLI from sun | Extra DLI to complete literature value | Extra DLI to complete carryover effect | DLI from sun | Extra DLI to complete literature value | Extra DLI to complete carryover effect | DLI from sun | Extra DLI to complete literature value | Extra DLI to complete carryover effect |
| 1 | 22.5 | 0 | 0 | 22.5 | 0 | 0 | 22.5 | 0 | 0 | 22.5 | 0 | 0 |
| 2 | 11.25 | 3.75 | 0 | 12.5 | 2.5 | 0 | 13.3 | 1.7 | 0 | 13.5 | 1.5 | 0 |
| 3 | 11.25 | 3.75 | 0 | 12.5 | 2.5 | 0 | 13.3 | 1.7 | 0 | 13.5 | 1.5 | 0 |
| 4 | 22.5 | 0 | 0 | 12.5 | 2.5 | 0 | 13.3 | 1.7 | 0 | 13.5 | 1.5 | 0 |
| 5 | 11.25 | 3.75 | 0 | 22.5 | 0 | 0 | 13.3 | 1.7 | 0 | 13.5 | 1.5 | 0 |
| 6 | 11.25 | 3.75 | 0 | 12.5 | 2.5 | 0 | 22.5 | 0 | 0 | 13.5 | 1.5 | 0 |
| 7 | 22.5 | 0 | 0 | 12.5 | 2.5 | 0 | 13.3 | 1.7 | 0 | 22.5 | 0 | 0 |
| 8 | 11.25 | 3.75 | 0 | 12.5 | 2.5 | 0 | 13.3 | 1.7 | 0 | 13.5 | 1.5 | 0 |
| 9 | 11.25 | 3.75 | 0 | 22.5 | 0 | 0 | 13.3 | 1.7 | 0 | 13.5 | 1.5 | 0 |
| 10 | 22.5 | 0 | 0 | 12.5 | 2.5 | 0 | 13.3 | 1.7 | 0 | 13.5 | 1.5 | 0 |
| 11 | 11.25 | 3.75 | 0 | 12.5 | 2.5 | 0 | 22.5 | 0 | 0 | 13.5 | 1.5 | 0 |
| 12 | 11.25 | 3.75 | 0 | 12.5 | 2.5 | 0 | 13.3 | 1.7 | 0 | 13.5 | 1.5 | 0 |
| 13 | 22.5 | 0 | 0 | 22.5 | 0 | 0 | 13.3 | 1.7 | 0 | 22.5 | 0 | 0 |
| 14 | 11.25 | 3.75 | 0 | 12.5 | 2.5 | 0 | 13.3 | 1.7 | 0 | 13.5 | 1.5 | 0 |
| 15 | 11.25 | 3.75 | 0 | 12.5 | 2.5 | 0 | 13.3 | 1.7 | 0 | 13.5 | 1.5 | 0 |
| 16 | 22.5 | 0 | 0 | 12.5 | 2.5 | 0 | 22.5 | 0 | 0 | 13.5 | 1.5 | 0 |
| 17 | 11.25 | 3.75 | 0 | 22.5 | 0 | 0 | 13.3 | 1.7 | 0 | 13.5 | 1.5 | 0 |
| 18 | 11.25 | 3.75 | 0 | 12.5 | 2.5 | 0 | 13.3 | 1.7 | 0 | 13.5 | 1.5 | 0 |
| 19 | 22.5 | 0 | 0 | 12.5 | 2.5 | 0 | 13.3 | 1.7 | 0 | 22.5 | 0 | 0 |
| 20 | 11.25 | 3.75 | 0 | 12.5 | 2.5 | 0 | 13.3 | 1.7 | 0 | 13.5 | 1.5 | 0 |
| 21 | 11.25 | 3.75 | 0 | 22.5 | 0 | 0 | 22.5 | 0 | 0 | 13.5 | 1.5 | 0 |
| 22 | 22.5 | 0 | 0 | 12.5 | 2.5 | 0 | 13.3 | 1.7 | 0 | 13.5 | 1.5 | 0 |
| 23 | 11.25 | 3.75 | 0 | 12.5 | 2.5 | 0 | 13.3 | 1.7 | 0 | 13.5 | 1.5 | 0 |
| 24 | 11.25 | 3.75 | 0 | 12.5 | 2.5 | 0 | 13.3 | 1.7 | 0 | 13.5 | 1.5 | 0 |
| 25 | 22.5 | 0 | 0 | 22.5 | 0 | 0 | 13.3 | 1.7 | 0 | 22.5 | 0 | 0 |
| 26 | 11.25 | 3.75 | 0 | 12.5 | 2.5 | 0 | 22.5 | 0 | 0 | 13.5 | 1.5 | 0 |
| 27 | 11.25 | 3.75 | 0 | 12.5 | 2.5 | 0 | 13.3 | 1.7 | 0 | 13.5 | 1.5 | 0 |
| 28 | 22.5 | 0 | 0 | 12.5 | 2.5 | 0 | 13.3 | 1.7 | 0 | 13.5 | 1.5 | 0 |
| 29 | 11.25 | 3.75 | 0 | 22.5 | 0 | 0 | 13.3 | 1.7 | 0 | 13.5 | 1.5 | 0 |
| 30 | 11.25 | 3.75 | 0 | 12.5 | 2.5 | 0 | 13.3 | 1.7 | 0 | 13.5 | 1.5 | 0 |
| 31 | 22.5 | 0 | 0 | 12.5 | 2.5 | 0 | 22.5 | 0 | 0 | 22.5 | 0 | 0 |
| 32 | 11.25 | 3.75 | 0 | 12.5 | 2.5 | 0 | 13.3 | 1.7 | 0 | 13.5 | 1.5 | 0 |
| 33 | 11.25 | 3.75 | 0 | 22.5 | 0 | 0 | 13.3 | 1.7 | 0 | 13.5 | 1.5 | 0 |
| 34 | 22.5 | 0 | 0 | 12.5 | 2.5 | 0 | 13.3 | 1.7 | 0 | 13.5 | 1.5 | 0 |
| 35 | 11.25 | 3.75 | 0 | 12.5 | 2.5 | 0 | 13.3 | 1.7 | 0 | 13.5 | 1.5 | 0 |
| 36 | 11.25 | 3.75 | 0 | 12.5 | 2.5 | 0 | 22.5 | 0 | 0 | 13.5 | 1.5 | 0 |
| 37 | 22.5 | 0 | 0 | 22.5 | 0 | 0 | 13.3 | 1.7 | 0 | 22.5 | 0 | 0 |
| 38 | 11.25 | 3.75 | 0 | 12.5 | 2.5 | 0 | 13.3 | 1.7 | 0 | 13.5 | 1.5 | 0 |
| 39 | 11.25 | 3.75 | 0 | 12.5 | 2.5 | 0 | 13.3 | 1.7 | 0 | 13.5 | 1.5 | 0 |
| 40 | 22.5 | 0 | 0 | 12.5 | 2.5 | 0 | 13.3 | 1.7 | 0 | 13.5 | 1.5 | 0 |
| 41 | 11.25 | 3.75 | 0 | 22.5 | 0 | 0 | 22.5 | 0 | 0 | 13.5 | 1.5 | 0 |
| 42 | 11.25 | 3.75 | 0 | 12.5 | 2.5 | 0 | 13.3 | 1.7 | 0 | 13.5 | 1.5 | 0 |
| 43 | 22.5 | 0 | 0 | 12.5 | 2.5 | 0 | 13.3 | 1.7 | 0 | 22.5 | 0 | 0 |
| 44 | 11.25 | 3.75 | 0 | 12.5 | 2.5 | 0 | 13.3 | 1.7 | 0 | 13.5 | 1.5 | 0 |
| 45 | 11.25 | 3.75 | 0 | 22.5 | 0 | 0 | 13.3 | 1.7 | 0 | 13.5 | 1.5 | 0 |
| 46 | 22.5 | 0 | 0 | 12.5 | 2.5 | 0 | 22.5 | 0 | 0 | 13.5 | 1.5 | 0 |
| 47 | 11.25 | 3.75 | 0 | 12.5 | 2.5 | 0 | 13.3 | 1.7 | 0 | 13.5 | 1.5 | 0 |
| 48 | 11.25 | 3.75 | 0 | 12.5 | 2.5 | 0 | 13.3 | 1.7 | 0 | 13.5 | 1.5 | 0 |
| 49 | 22.5 | 0 | 0 | 22.5 | 0 | 0 | 13.3 | 1.7 | 0 | 22.5 | 0 | 0 |
| 50 | 11.25 | 3.75 | 0 | 12.5 | 2.5 | 0 | 13.3 | 1.7 | 0 | 13.5 | 1.5 | 0 |
| 51 | 11.25 | 3.75 | 0 | 12.5 | 2.5 | 0 | 22.5 | 0 | 0 | 13.5 | 1.5 | 0 |
| 52 | 22.5 | 0 | 0 | 12.5 | 2.5 | 0 | 13.3 | 1.7 | 0 | 13.5 | 1.5 | 0 |
| 53 | 11.25 | 3.75 | 0 | 22.5 | 0 | 0 | 13.3 | 1.7 | 0 | 13.5 | 1.5 | 0 |
| 54 | 11.25 | 3.75 | 0 | 12.5 | 2.5 | 0 | 13.3 | 1.7 | 0 | 13.5 | 1.5 | 0 |
| 55 | 22.5 | 0 | 0 | 12.5 | 2.5 | 0 | 13.3 | 1.7 | 0 | 22.5 | 0 | 0 |
| 56 | 11.25 | 3.75 | 0 | 12.5 | 2.5 | 0 | 22.5 | 0 | 0 | 13.5 | 1.5 | 0 |
| 57 | 11.25 | 3.75 | 0 | 22.5 | 0 | 0 | 13.3 | 1.7 | 0 | 13.5 | 1.5 | 0 |
| 58 | 22.5 | 0 | 0 | 12.5 | 2.5 | 0 | 13.3 | 1.7 | 0 | 13.5 | 1.5 | 0 |
| 59 | 11.25 | 3.75 | 0 | 12.5 | 2.5 | 0 | 13.3 | 1.7 | 0 | 13.5 | 1.5 | 0 |
| 60 | 11.25 | 3.75 | 0 | 12.5 | 2.5 | 0 | 13.3 | 1.7 | 0 | 13.5 | 1.5 | 0 |

Supplemental table 2. Lighting regime for the second hypothetical case to asses energy requirement depending on the number of days with low DLI after a day with high DLI. Lines in yellow indicate the day with high DLI, and lines in white indicate days with low DLI. For each combination of days with low DLI after a day with high DLI (lighting regime), we indicate the DLI received from the sun, the extra DLI to be achieved, the DLI reported in the literature for optimal plant growth, and the extra DLI to complete a DLI to take advantage of the ‘carryover’ effect.

|  | Lighting regime | | | | | | | | | | | |
| --- | --- | --- | --- | --- | --- | --- | --- | --- | --- | --- | --- | --- |
|  | 2 days with low DLI | | | 3 days with low DLI | | | 4 days with low DLI | | | 5 days with low DLI | | |
| Days | DLI from sun | Extra DLI to complete literature value | Extra DLI to complete carryover effect | DLI from sun | Extra DLI to complete literature value | Extra DLI to complete carryover effect | DLI from sun | Extra DLI to complete literature value | Extra DLI to complete carryover effect | DLI from sun | Extra DLI to complete literature value | Extra DLI to complete carryover effect |
| 1 | 22.5 | 0 | 0 | 22.5 | 0 | 0 | 22.5 | 0 | 0 | 22.5 | 0 | 0 |
| 2 | 10 | 5 | 1.25 | 10 | 5 | 2.5 | 10 | 5 | 3.13 | 10 | 5 | 3.5 |
| 3 | 10 | 5 | 1.25 | 10 | 5 | 2.5 | 10 | 5 | 3.13 | 10 | 5 | 3.5 |
| 4 | 22.5 | 0 | 0 | 10 | 5 | 2.5 | 10 | 5 | 3.13 | 10 | 5 | 3.5 |
| 5 | 10 | 5 | 1.25 | 22.5 | 0 | 0 | 10 | 5 | 3.13 | 10 | 5 | 3.5 |
| 6 | 10 | 5 | 1.25 | 10 | 5 | 2.5 | 22.5 | 0 | 0 | 10 | 5 | 3.5 |
| 7 | 22.5 | 0 | 0 | 10 | 5 | 2.5 | 10 | 5 | 3.13 | 22.5 | 0 | 0 |
| 8 | 10 | 5 | 1.25 | 10 | 5 | 2.5 | 10 | 5 | 3.13 | 10 | 5 | 3.5 |
| 9 | 10 | 5 | 1.25 | 22.5 | 0 | 0 | 10 | 5 | 3.13 | 10 | 5 | 3.5 |
| 10 | 22.5 | 0 | 0 | 10 | 5 | 2.5 | 10 | 5 | 3.13 | 10 | 5 | 3.5 |
| 11 | 10 | 5 | 1.25 | 10 | 5 | 2.5 | 22.5 | 0 | 0 | 10 | 5 | 3.5 |
| 12 | 10 | 5 | 1.25 | 10 | 5 | 2.5 | 10 | 5 | 3.13 | 10 | 5 | 3.5 |
| 13 | 22.5 | 0 | 0 | 22.5 | 0 | 0 | 10 | 5 | 3.13 | 22.5 | 0 | 0 |
| 14 | 10 | 5 | 1.25 | 10 | 5 | 2.5 | 10 | 5 | 3.13 | 10 | 5 | 3.5 |
| 15 | 10 | 5 | 1.25 | 10 | 5 | 2.5 | 10 | 5 | 3.13 | 10 | 5 | 3.5 |
| 16 | 22.5 | 0 | 0 | 10 | 5 | 2.5 | 22.5 | 0 | 0 | 10 | 5 | 3.5 |
| 17 | 10 | 5 | 1.25 | 22.5 | 0 | 0 | 10 | 5 | 3.13 | 10 | 5 | 3.5 |
| 18 | 10 | 5 | 1.25 | 10 | 5 | 2.5 | 10 | 5 | 3.13 | 10 | 5 | 3.5 |
| 19 | 22.5 | 0 | 0 | 10 | 5 | 2.5 | 10 | 5 | 3.13 | 22.5 | 0 | 0 |
| 20 | 10 | 5 | 1.25 | 10 | 5 | 2.5 | 10 | 5 | 3.13 | 10 | 5 | 3.5 |
| 21 | 10 | 5 | 1.25 | 22.5 | 0 | 0 | 22.5 | 0 | 0 | 10 | 5 | 3.5 |
| 22 | 22.5 | 0 | 0 | 10 | 5 | 2.5 | 10 | 5 | 3.13 | 10 | 5 | 3.5 |
| 23 | 10 | 5 | 1.25 | 10 | 5 | 2.5 | 10 | 5 | 3.13 | 10 | 5 | 3.5 |
| 24 | 10 | 5 | 1.25 | 10 | 5 | 2.5 | 10 | 5 | 3.13 | 10 | 5 | 3.5 |
| 25 | 22.5 | 0 | 0 | 22.5 | 0 | 0 | 10 | 5 | 3.13 | 22.5 | 0 | 0 |
| 26 | 10 | 5 | 1.25 | 10 | 5 | 2.5 | 22.5 | 0 | 0 | 10 | 5 | 3.5 |
| 27 | 10 | 5 | 1.25 | 10 | 5 | 2.5 | 10 | 5 | 3.13 | 10 | 5 | 3.5 |
| 28 | 22.5 | 0 | 0 | 10 | 5 | 2.5 | 10 | 5 | 3.13 | 10 | 5 | 3.5 |
| 29 | 10 | 5 | 1.25 | 22.5 | 0 | 0 | 10 | 5 | 3.13 | 10 | 5 | 3.5 |
| 30 | 10 | 5 | 1.25 | 10 | 5 | 2.5 | 10 | 5 | 3.13 | 10 | 5 | 3.5 |
| 31 | 22.5 | 0 | 0 | 10 | 5 | 2.5 | 22.5 | 0 | 0 | 22.5 | 0 | 0 |
| 32 | 10 | 5 | 1.25 | 10 | 5 | 2.5 | 10 | 5 | 3.13 | 10 | 5 | 3.5 |
| 33 | 10 | 5 | 1.25 | 22.5 | 0 | 0 | 10 | 5 | 3.13 | 10 | 5 | 3.5 |
| 34 | 22.5 | 0 | 0 | 10 | 5 | 2.5 | 10 | 5 | 3.13 | 10 | 5 | 3.5 |
| 35 | 10 | 5 | 1.25 | 10 | 5 | 2.5 | 10 | 5 | 3.13 | 10 | 5 | 3.5 |
| 36 | 10 | 5 | 1.25 | 10 | 5 | 2.5 | 22.5 | 0 | 0 | 10 | 5 | 3.5 |
| 37 | 22.5 | 0 | 0 | 22.5 | 0 | 0 | 10 | 5 | 3.13 | 22.5 | 0 | 0 |
| 38 | 10 | 5 | 1.25 | 10 | 5 | 2.5 | 10 | 5 | 3.13 | 10 | 5 | 3.5 |
| 39 | 10 | 5 | 1.25 | 10 | 5 | 2.5 | 10 | 5 | 3.13 | 10 | 5 | 3.5 |
| 40 | 22.5 | 0 | 0 | 10 | 5 | 2.5 | 10 | 5 | 3.13 | 10 | 5 | 3.5 |
| 41 | 10 | 5 | 1.25 | 22.5 | 0 | 0 | 22.5 | 0 | 0 | 10 | 5 | 3.5 |
| 42 | 10 | 5 | 1.25 | 10 | 5 | 2.5 | 10 | 5 | 3.13 | 10 | 5 | 3.5 |
| 43 | 22.5 | 0 | 0 | 10 | 5 | 2.5 | 10 | 5 | 3.13 | 22.5 | 0 | 0 |
| 44 | 10 | 5 | 1.25 | 10 | 5 | 2.5 | 10 | 5 | 3.13 | 10 | 5 | 3.5 |
| 45 | 10 | 5 | 1.25 | 22.5 | 0 | 0 | 10 | 5 | 3.13 | 10 | 5 | 3.5 |
| 46 | 22.5 | 0 | 0 | 10 | 5 | 2.5 | 22.5 | 0 | 0 | 10 | 5 | 3.5 |
| 47 | 10 | 5 | 1.25 | 10 | 5 | 2.5 | 10 | 5 | 3.13 | 10 | 5 | 3.5 |
| 48 | 10 | 5 | 1.25 | 10 | 5 | 2.5 | 10 | 5 | 3.13 | 10 | 5 | 3.5 |
| 49 | 22.5 | 0 | 0 | 22.5 | 0 | 0 | 10 | 5 | 3.13 | 22.5 | 0 | 0 |
| 50 | 10 | 5 | 1.25 | 10 | 5 | 2.5 | 10 | 5 | 3.13 | 10 | 5 | 3.5 |
| 51 | 10 | 5 | 1.25 | 10 | 5 | 2.5 | 22.5 | 0 | 0 | 10 | 5 | 3.5 |
| 52 | 22.5 | 0 | 0 | 10 | 5 | 2.5 | 10 | 5 | 3.13 | 10 | 5 | 3.5 |
| 53 | 10 | 5 | 1.25 | 22.5 | 0 | 0 | 10 | 5 | 3.13 | 10 | 5 | 3.5 |
| 54 | 10 | 5 | 1.25 | 10 | 5 | 2.5 | 10 | 5 | 3.13 | 10 | 5 | 3.5 |
| 55 | 22.5 | 0 | 0 | 10 | 5 | 2.5 | 10 | 5 | 3.13 | 22.5 | 0 | 0 |
| 56 | 10 | 5 | 1.25 | 10 | 5 | 2.5 | 22.5 | 0 | 0 | 10 | 5 | 3.5 |
| 57 | 10 | 5 | 1.25 | 22.5 | 0 | 0 | 10 | 5 | 3.13 | 10 | 5 | 3.5 |
| 58 | 22.5 | 0 | 0 | 10 | 5 | 2.5 | 10 | 5 | 3.13 | 10 | 5 | 3.5 |
| 59 | 10 | 5 | 1.25 | 10 | 5 | 2.5 | 10 | 5 | 3.13 | 10 | 5 | 3.5 |
| 60 | 10 | 5 | 1.25 | 10 | 5 | 2.5 | 10 | 5 | 3.13 | 10 | 5 | 3.5 |
